# Proteomic Analysis of Hippocampus in a Mouse Model of Depression Reveals Neuroprotective Function of Ubiquitin C-terminal Hydrolase L1 (UCH-L1) via Stress-induced Cysteine Oxidative Modifications*[Fn FN2]

**DOI:** 10.1074/mcp.RA118.000835

**Published:** 2018-06-29

**Authors:** Jung-Eun Choi, Jae-Jin Lee, Wonmo Kang, Hyun Jung Kim, Jin-Hwan Cho, Pyung-Lim Han, Kong-Joo Lee

**Affiliations:** From the ‡College of Pharmacy and Graduate School of Pharmaceutical Sciences, and; §Department of Brain and Cognitive Sciences, Ewha Womans University, Seoul, Korea 03760

**Keywords:** Protein Modification, Deubiquitinases, Oxidative stress, Mouse models, Ubiquitin, Tandem Mass Spectrometry, Thiol redox chemistry, Neurobiology, chronic restraint stress, depression model, hippocampus

## Abstract

Chronic physical restraint stress increases oxidative stress in the brain, and dysregulation of oxidative stress can be one of the causes of major depressive disorder. To understand the underlying mechanisms, we undertook a systematic proteomic analysis of hippocampus in a chronic restraint stress mouse model of depression. Combining two-dimensional gel electrophoresis (2D-PAGE) for protein separation with nanoUPLC-ESI-q-TOF tandem mass spectrometry, we identified sixty-three protein spots that changed in the hippocampus of mice subjected to chronic restraint stress. We identified and classified the proteins that changed after chronic stress, into three groups respectively functioning in neural plasticity, metabolic processes and protein aggregation. Of these, 5 proteins including ubiquitin C-terminal hydrolase L1 (UCH-L1), dihydropyrimidinase-related protein 2 (DPYL2), haloacid dehalogenase-like hydrolase domain-containing protein 2 (HDHD2), actin-related protein 2/3 complex subunit 5 (ARPC5) and peroxiredoxin-2 (PRDX2), showed pI shifts attributable to post-translational modifications. Further analysis indicated that UCH-L1 underwent differential oxidations of 2 cysteine residues following chronic stress. We investigated whether the oxidized form of UCH-L1 plays a role in stressed hippocampus, by comparing the effects of UCH-L1 and its Cys mutants on hippocampal cell line HT-22 in response to oxidative stress. This study demonstrated that UCH-L1 wild-type and cysteine to aspartic acid mutants, but not its cysteine to serine mutants, afforded neuroprotective effects against oxidative stress; there were no discernible differences between wild-type UCH-L1 and its mutants in the absence of oxidative stress. These findings suggest that cysteine oxidative modifications of UCH-L1 in the hippocampus play key roles in neuroprotection against oxidative stress caused in major depressive disorder.

Major depressive disorder (MDD)^1^ in human is a life-threatening mood disorder, accompanied, in addition to persistent depression, by lethargy, and memory impairment among other symptoms. Because stress is generally accepted as one of the main causes of MDD (1), several animal models of depression have been developed. One of these is the chronic restraint stress (CRS)-induced depression mouse model. In this model, the hippocampus is reduced in size along with neurite deterioration (2, 3). Previous studies with depression models have reported a relationship between stress and neural plasticity (4). cAMP-PKA-CREB, MAPK, and Akt-mTOR signaling pathways, in particular, were found to be involved in the stress-induced changes in synaptic plasticity (5). Chronic restraint stress (CRS) mouse model is produced when mice are restrained for 2 h daily for 14 days, characterized by lasting anxiety- and depression-like behaviors, and induces changes in synaptic plasticity at the gene level (6). In this model, oxidative stress because of upregulation of NADPH oxidase occurs in the hippocampus (7). Moreover, recent meta-analysis studies have suggested the role of oxidative stress in MDD patients (8, 9). Proteomic approaches used in most of previous studies to identify the molecular changes in hippocampus of various rodent depression models, have focused on the genes or proteins whose expression levels are changed after stress (10, 11). However, additional validation of identified targets in depression is needed by identifying post-translational modifications (PTMs), because PTMs regulate cellular functions of proteins.

Ubiquitin C-terminal hydrolase isozyme L1 (UCH-L1) is an enzyme that hydrolyzes small C-terminal adducts of ubiquitin and generates ubiquitin monomer (12). UCH-L1 is expressed in all neurons and is one of the most abundant proteins in the brain (13). UCH-L1 is essential for normal synaptic and cognitive memory function (14). UCH-L1 also functions as an antioxidant, and it undergoes extensive oxidative modifications in neurodegenerative diseases such as Alzheimer's disease (AD) and Parkinson's disease (PD) (15). We previously determined the redox sensitivities of Cys90 and Cys152 in UCH-L1 using the chemical probe, biotin labeled methyl-3-nitro-4-(piperidin-1-ylsulfonyl) benzoate (NPSB-B), that selectively and specifically reacts with sulfhydryl of redox-sensitive Cys residues (16). Although the active site of UCH-L1 for its hydrolase activity is Cys90, covalent modifications at Cys152 under oxidative stress or inflammatory response are associated with the disruption of its native structure, loss of solubility, and aggregation behavior, resulting in cytotoxicity (17–19). However, the molecular function or the effects of modifications of UCH-L1 in depressive brains have not been studied.

In the present study, using 2D-PAGE-based proteomic analysis, we identified sixty-three stress-responsive proteins in the hippocampus of chronic restraint stress mouse model and found the most significant changes in UCH-L1. We focused on oxidative modifications of Cys of UCH-L1 and suggest that oxidative regulation of UCH-L1 plays a key role in neuroprotection in major depressive disorder. We further demonstrated that the overexpression of wild-type (WT) UCH-L1 in HT-22 hippocampal cells afforded protective effects against oxidative stress, whereas its Cys to Ser mutants abolished this effect and Cys to Asp mutants behaved like WT. These results reveal a novel neuroprotective function of UCH-L1 via stress-induced Cys oxidative modifications.

## EXPERIMENTAL PROCEDURES

### 

#### 

##### Animals, Chronic Restraint Stress Treatment, and Tissue Sample Preparation

Chronic restraint stress mouse model was produced as described previously (6). Six-week-old male C57BL/6 mice were purchased from the Central Lab. Animal Inc. (Seoul, Korea), and were randomly separated into four groups: those exposed to stress for 9 days and their controls (2 groups) and those exposed to stress for 14 days and their controls (2 groups). All groups of mice were given free access to food and water during the entire experiment. For restraint stress treatment, mice were individually placed and firmly restrained in a well-ventilated 50 ml conical tube for 2 h daily and returned to their original cages, and this procedure was repeated for 9 or 14 consecutive days. Stressed and unstressed groups were kept spatially separated. All experiments were approved by Ewha Womans University IACUC (IACUC No. 2012-01-049).

On the last day of stress treatment, the animals were anesthetized with intraperitoneal injections of Zoletil 50 (50 mg/kg; Virbac, Carros, France) and Rompun (10 mg/kg; Bayer, Leverkusen, Germany) and transcardially perfused with chilled heparin/PBS solution (10 units/ml; JW Pharmaceutical, Seoul, Korea) using a 20 ml syringe to remove red blood cells in the tissue. Whole mouse brains were separated, and the hippocampi were dissected using a needle of 1 ml syringe under microscopic view. Tissue samples were stored in a −80 °C deep freezer until use for proteomic analysis.

##### Antibodies and Materials

Antibodies used in this study were purchased from following manufacturers: anti-UCH-L1 (Millipore, Burlington, MA), anti-DPYL2 (Cell Signaling Technology, Danvers, MA), anti-ARPC5 (Abcam, Cambridge, UK), anti-PRDX2 (Ab Frontier, Seoul, Korea), anti-Myc (Millipore Waltham, MA) and anti-tubulin (Santa Cruz Biotechnology, Dallas, TX). Anti-HDHD2 antibody was custom-made from AbClon (Seoul, Korea). Dulbecco's Modified Eagle Medium (DMEM), F-12 medium, Opti-MEM, FBS, trypsin and Pen Strep for HT-22 cell culture were purchased from Gibco Life Technologies (Waltham, MA), Eagle's Minimum Essential Medium (EMEM) was from WELGENE (Gyeongsan, Korea), EDTA, DTT, and Tris from Duchefa (Haarlem, The Netherlands), SDS from Amresco (Solon, OH), and all other chemicals used in proteomic analysis including HEPES, NaCl, KCl, Triton X-100, glycerol, protease inhibitor mixture, Na_3_VO_4_, trichostatin A, sodium butyrate, PMSF, acetone, urea, iodoacetamide, bromphenol blue, ACN, formic acid, methanol, ethanol, xylene, and bis-acrylamide from Sigma-Aldrich (St. Louis, MO) unless otherwise mentioned.

##### Two-dimensional Polyacrylamide Gel Electrophoresis (2D-PAGE) and Image Analysis

To compare the protein expression profiles of control and stressed mice and to study specifically post-translationally modified proteins, we employed 2D-PAGE for protein separation. Whole hippocampal protein extracts from each sample were obtained by grinding the tissue with liquid nitrogen and lysing it with lysis buffer (20 mm HEPES, pH 7.4, 50 mm NaCl, 40 mm KCl, 1% (w/v) Triton X-100, 10% (w/v) glycerol, 0.5% (v/v) protease inhibitor mixture, 2.5 mm Na_3_VO_4_, 5 mm trichostatin A, 5 mm sodium butyrate, 1 mm PMSF, 1 mm EDTA). The lysates were centrifuged at 20,000 × *g* for 30 min at 4 °C. The supernatants were collected and centrifuged again under the same condition. Protein concentration of each sample was determined using BCA assay kit (Pierce, Waltham, MA). Proteins were precipitated by adding acetone containing 1% DTT to cell lysate, vortexing the tube and incubating samples at −20 °C for 1 h. After centrifugation at 10,000 × *g* for 5 min at 4 °C, the pellets were collected and dissolved in tissue lysis buffer by incubating at room temperature for 2 h with vortexing at 30 min intervals. Proteins (150 μg) of each sample were loaded on a rehydrated strip gels (BIO-RAD (Hercules, CA), 18 cm, pH 4–7). 2D-PAGE was performed as previously described (20). IEF was carried out using Ettan IPGphor II isoelectric focusing unit (Amersham Bioscience, Little Chalfont, UK) and gel strips were equilibrated in two steps using the first equilibration buffer (50 mm Tris-Cl, pH 8.8, 6 m urea, 2% (w/v) SDS, 30% (w/v) glycerol) added with 65 mm DTT and the second equilibration buffer supplemented with 2.5% (w/v) iodoacetamide and bromphenol blue. SDS-PAGE was performed using PROTEAN II xi 2-D cell apparatus (BIO-RAD) and all gels were simultaneously stained by silver staining method in the same tray.

For image analysis, gel images were obtained using Image Scanner III (GE Healthcare, Chicago, IL). Progenesis SameSpots (version 5.0, Nonlinear Dynamics, Newcastle upon Tyne UK) auto-processing 2D-PAGE gel analysis software was used for gel image alignment, normalization, spot detection, quantification of spot intensities and statistical analysis. Spots showing at least 1.3-fold difference in three replicates of each group (with a *p* value of less than 0.05) were considered statistically significant and were targeted for protein identification.

##### Protein Detection with nanoUPLC-ESI-q-TOF Tandem MS

The gel spots of differentially expressed proteins were identified by peptide sequencings employing nanoAcquity™ UPLC™-ESI-Q-TOF mass spectrometry (SYNAPT™ G2-Si™, Waters Co., Milford, MA) as previously described with a few modifications (21). Modifications specifically applied for this study are as follows; peptides were eluted with a linear gradient of 5–40% buffer B (ACN/formic acid; 100: 0.1, v/v) with buffer A (water/formic acid; 100: 0.1, v/v) over 80 min and MS scan cycle was composed of one MS scan followed by MS/MS scans of the 10 most abundant ions in each MS scan.

##### Search Parameters and Acceptance Criteria for MS Data

For further processing of raw mass spectrometry data, peaklist files (.pkl) were generated using Protein-Lynx Global Server (PLGS) 2.3 data processing software (Waters Co.). Peaklists were searched against protein sequence databases NCBInr (release date 20151106, 74513707 entries) and SwissProt (version 51.6, 257964 entries) using a global search engine Mascot (version 2.2.0, Boston, MA, USA). Taxonomy filter for 2D-PAGE samples was *Mus musculus* (house mouse) and *Homo sapiens* (human) for overexpressed UCH-L1 samples (supplemental Fig. 4). Trypsin was the only protease used to generate peptides. Maximum number of 1 missed cleavage was permitted. No fixed modifications were considered. As variable modifications, acetylation and formylation of Lys, N-terminal pyroglutamylation of Gln and Glu, oxidation of Met, carbamidomethylation of Cys, acrylamide adduct propionamide of Cys and phosphorylation of Ser or Thr were considered for identification of 2D-PAGE protein spots. For PTM analysis, we employed SEMSA (22) methodology to achieve higher MS sequence coverage and MOD^i^ (Korea, http://prix.hanyang.ac.kr/) PTM searching algorithm (23). For Cys oxidative modification search of hippocampus samples, oxidation, dioxidation, and trioxidation of Cys, Cys-SO_2_-SH, conversion of Cys to Ser, and chemical adduct carbamidomethylation of Cys were used as variable modifications. Phosphorylation of Ser, Thr, and Tyr, acetylation of Lys, deamidation of Asn and Gln, oxidation of Met, methylation of Arg, propionamide of Cys, and nitrosylation of Cys were also used as variable modifications in combination with the modifications stated above to search unknown PTMs. As for overexpressed UCH-L1 samples, oxidation of Met, phosphorylation of Ser or Thr, oxidation, dioxidation and trioxidation of Cys, conversion of Cys to Ser, Cys-SO_2_-SH, acetylation of Lys, and carbamidomethylation of Cys were considered as variable modifications. Mass tolerance for precursor ions was ± 0.5 Da and mass tolerance for fragment ions was ± 0.5 Da because the error value of MS was about 0.01. For interpretation of search results, keratin was excluded as a known contaminant from sample preparation. Significant matches were sorted by the threshold indicated by Mascot probability analysis and matches with probability-based Mowse score *p* < 0.05 (and expectation value of 0.05) were considered significant. Ions score was calculated as −10 x Log(P) (P: the probability that the observed match is a random event) and automatically provided as Mascot result. Regarding the protein identification result, the best match with the highest score among identified results was used as the identification result for the spot. All spectra were manually verified to sort out meaningful results. For PTM search results, modified peptides with minimum total mascot score 30 was considered good enough for PTM analysis, but if the same peptide with the same modification was found in other spots, the result was included even if the score was lower than 30 for comparison. PTM results were verified not just by score threshold, but by checking each spectrum carefully. Other PTMs with low scores were presented in supplemental Table S1 for ambiguous PTMs. In the case of peptides that could not be annotated accurately, they were classified as ambiguous PTMs (supplemental Table S1) even if the scores were high.

##### Protein Network and Functional Analysis of Identified Proteins

The identified proteins were classified into subgroups based on their biological processes and molecular functions employing text-mining and STRING protein network analysis program (24). The functions of identified proteins were determined using the PANTHER classification system (www.pantherdb.org) (25).

##### Western Blot Analysis

Hippocampal proteins from control and stressed mice were separated by mini 2D-PAGE and transferred to hydrophobic PDVF membrane (Millipore). Distributions of UCH-L1, DPYL2, HDHD2, ARPC5, and PRDX2 spots were verified by Western blotting using each antibody. Amersham Biosciences ECL Prime Western blotting detection reagent (GE Healthcare) and LAS-3000 imaging system (Fujifilm, Tokyo, Japan) was used to gain chemiluminescent signals and Multi Gauge V3.0 (Fujifilm) was used for image analysis. For 1D Western blot analysis performed in HT-22 cell experiment, 5 × 10^5^ cells were lysed in 75 μl of SDS gel sample buffer. The amount of overexpressed UCH-L1 and tubulin was detected using anti-Myc and anti-tubulin antibodies. Anti-UCH-L1 antibody was used to detect endogenous UCH-L1.

##### Immunohistochemistry

Paraffin-embedded coronal sections of mouse brains were kindly provided by Professor Kyunglim Lee (Ewha Womans University). Brain sections were deparaffinized by incubating slides in Xylene, three times for 10 min each, and washing with 100% ethanol three times for 5 min each. Endogenous peroxidase quenching was performed with quenching solution (H_2_O_2_/Methanol; 3:200, v/v) for 15 min. Following epitope retrieval and immunostaining were conducted using anti-UCH-L1 antibody (Millipore #Ab1761-I) and Bethyl immunohistochemistry accessory kit (#IHC-101, Bethyl Laboratories Inc., Montgomery, TX) according to the manufacturers' instructions. In brief, brain sections were incubated in 96 °C with retrieval buffer for 20 min to retrieve epitopes. After treating with blocking solution for 15 min at room temperature, brain sections were incubated overnight at 4 °C with anti-UCH-L1 (1:100) or antibody diluent solution (for negative control). Anti-rabbit secondary antibody and 3,3′-diaminobenzidine (DAB) staining for 1 min were used to detect UCH-L1. Hematoxylin counterstaining for 2 min was used to stain cell nuclei. Slides were then treated with IHC-Bluing solution, dehydrated in ethanol, cleared in xylene and covered with coverslip using permanent mounting media (Vector Laboratories, Burlingame, CA). Image analysis was performed using Axio Scope.A1 microscope (Carl Zeiss, Oberkochen, Germany) and AxioVision software version 4.9. (Carl Zeiss).

##### Cell Culture

HT-22 mouse hippocampal cell line used in this study was provided by Professor Hwa-Jung Kim (Ewha Womans University). Cells were maintained in high glucose Dulbecco's Modified Eagle Medium (DMEM) supplemented with 10% FBS, 100 units/ml penicillin and 100 μg/ml streptomycin (complete media, DMEM-CM). SH-SY5Y cells were purchased from ATCC. Cells were maintained in 1:1 mixture of Eagle's Minimum Essential Medium (EMEM) and F-12 medium supplemented with 10% FBS, 100 units/ml penicillin and 100 μg/ml streptomycin. All cells were incubated in a 37 °C incubator containing 5% CO_2_ in a humidified atmosphere.

##### Plasmids

Human UCH-L1 clone inserted into pcDNA3.1/myc-His(-) A vector (Invitrogen, Carlsbad, CA) was obtained as previously described (26). Cys to Ser mutants and Cys to Asp mutants of myc-tagged UCH-L1 were generated using QuikChange II Site-Directed Mutagenesis Kit (Agilent Technologies, Santa Clara, CA) according to the manufacturer's protocol. The primers used for mutagenesis are as follows: C90S_sense, 5′-GAC CAT TGG GAA TTC CAG TGG CAC AAT CGG ACT-3′; C90S_antisense, 5′-AGT CCG ATT GTG CCA CTG GAA TTC CCA ATG GTC-3′; C152S_sense, 5′-GTG GCA CAG GAA GGC CAA AGT CGG GTA GAT G-3′; C152S_antisense, 5′-CAT CTA CCC GAC TTT GGC CTT CCT GTG CCA C-3′; C90D_sense, 5′-GCA GAC CAT TGG GAA TTC CGA TGG CAC AAT CGG ACT TAT T-3′; C90D_antisense, 5′-AAT AAG TCC GAT TGT GCC ATC GGA ATT CCC AAT GGT CTG C-3′; C152D_sense, 5′-GCC GTG GCA CAG GAA GGC CAA GAT CGG GTA GAT G-3′; and C152D_antisense, 5′-CAT CTA CCC GAT CTT GGC CTT CCT GTG CCA CGG C-3′. Double mutants (C90/152S, C90/152D) were sequentially generated using single point-mutated DNA as a template. All plasmids were confirmed by DNA sequencing.

##### Transient Transfection

For transient overexpression of Myc-UCH-L1 and its Cys mutants, Lipofectamine 2000 (Invitrogen) reagent was used to transfect HT-22 cells in accordance with the manufacturer's protocol. Empty pcDNA3.1/myc-His(-) A vector was used as control plasmid and transfected to HT-22 cells in parallel with the plasmids carrying UCH-L1 DNA constructs. In brief, cells were plated on 60 mm culture plate 24 h before transfection and treated with 3 μg of plasmid and 7.5 μl of Lipofectamine 2000 reagent diluted in Opti-MEM. Cells were incubated for another 24 h before used for further analysis. For silencing UCH-L1 gene in SH-SY5Y cells, UCH-L1 small interfering RNA (siRNA) and control siRNA were obtained from Bioneer (Daejeon, Korea). Cells were transfected with Lipofectamine RNAiMAX reagent (Invitrogen) following the manufacturer's protocol at a final siRNA concentration of 100 nm. Cell medium was changed 6 h after transfection to reduce cell toxicity. Following WST-1 cell viability assay was performed 72 h post-transfection.

##### xCELLigence Real-time Cell Analysis (xCELLigence RTCA)

HT-22 cells were seeded at a density of 5 × 10^5^ cells/60-mm plate and grown for 24 h. Control plasmid pcDNA3.1/myc-His(-) A vector, and plasmids carrying wild-type (WT) and Cys mutants (C90S/D, C152S/D and C90/152S/D) of UCH-L1 were delivered into the cells respectively using Lipofectamine 2000 transfection reagent. At 24 h post-transfection, cells were washed twice with 1 ml Hanks' Balanced Salt Solution (HBSS) to remove serum. Cells were treated with 0 or 0.3 mm of H_2_O_2_ in HBSS for 2 h (37 °C, 5% CO_2_). Real-time cell growth was measured using xCELLigence RTCA instrument (ACEA Biosciences Inc., San Diego, CA). Briefly, 50 μl of DMEM-CM/well in 96-well RTCA E-plate was used to read the blank. After 2 h of H_2_O_2_ treatment, H_2_O_2_ was removed by washing cells sequentially with 1 ml HBSS and 1 ml PBS. Cells were trypsinized and 1 × 10^4^ cells in 100 μl DMEM-CM were added into the well containing 50 μl DMEM-CM. Cell index was monitored at 15 min intervals for 72 h. All samples were tested in triplicate.

##### WST-1 Cell Viability Assay

SH-SY5Y cells (7.5 × 10^5^ cells/60 mm plate) were plated and transfected with control and UCH-L1 siRNA at 24 h after seeding. At 48 h after transfection, cells were transferred to 96-well plate (1 × 10^5^ cells/well) and grown for 24 h. At 72 h after transfection, cells were treated with various concentrations of H_2_O_2_ (0, 0.1 and 0.25 mm) for 2 h at 37 °C in a 5% CO_2_ incubator as previously described. After 2 h, HBSS containing H_2_O_2_ was replaced with 50 μl culture medium and 5 μl WST-1 reagent (Roche Applied Science, Penzberg, Germany) per each well was added. Cells were incubated for 1 h in the incubator (37 °C, 5% CO_2_) and absorbances at 450 nm and 690 nm were measured using SpectraMax 190 Microplate Reader (Molecular Devices, San Jose, CA, USA) and SoftMax Pro 5.4.1 software (Molecular Devices). OD_450–690_ was used as cell viability index and data were normalized to viability of cells treated with 0 mm H_2_O_2_. UCH-L1 gene silencing was confirmed by Western blot analysis.

##### Experimental Design and Statistical Rationale

In this study, we used freshly isolated hippocampus respectively from unstressed controls (*n* = 6, consisting of three 9-day controls and three 14-day controls, which showed no significant difference) and stressed mice that were chronically restrained for 9 days (*n* = 3) or 14 days (*n* = 3). Two biological replicates were performed. 2D-PAGE was performed twice (set of 12 gels per experiment, one gel for each animal) in one animal experiment with varying amounts of proteins loaded and remaining samples were used for further verifications. Because chronic restraint stress causes extreme pain to the mouse, we used a minimal number of animals to receive IACUC approval. We chose three animals for each group because that is the minimum number needed for statistical analysis. Twelve is also the maximum number that can be used to minimize errors in the handling of 2D-PAGE procedure and the silver staining procedure which is performed in a single tray. Based on our previous study (6), 9 days of stress treatment was selected for examining the changes at early time point in hippocampus, whereas 14 days of stress was considered as completely depressed state. For protein identification of target proteins, MS analysis was performed twice, and three independent MS scans were performed for PTM analyses. For statistical analysis, Shapiro-Wilk test was applied as the normality test of all data sets. We chose Shapiro-Wilk test rather than Kolmogorov-Smirnov test because it is more suitable for small-sized sample group. Comparison of the two groups satisfying the normality was performed using a two-tailed Student's *t* test. Kruskal-Wallis test or Mann-Whitney *U* test was used to compare the means of two groups that did not satisfy the normality. For comparison of more than three groups, one-way ANOVA (analysis of variance) analysis was applied for samples satisfying normality and Kruskal-Wallis test (supported by pairwise comparisons using the Dunn-Bonferroni approach post-hoc test) was used for samples not satisfying the normality. Statistical analyses for 2D-PAGE spots were performed using Progenesis SameSpots (version 5.0, Nonlinear Dynamics) and other statistical analyses were performed using IBM SPSS Statistics (version 24, IBM, Armonk, NY).

## RESULTS

### 

#### 

##### Proteomic Changes in Hippocampus of CRS Model Mice, Following Chronic Restraint Stress

**Fig. 1.**
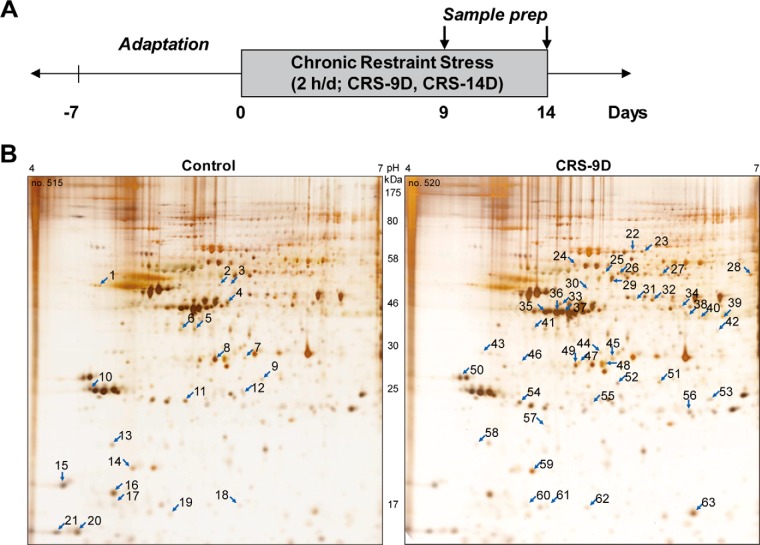
**Differential proteins in the hippocampus of CRS mouse identified by 2D-PAGE.** *A*, experimental design. Mice were restrained for 2 h daily for 9 or 14 days and tissue samples were prepared from sacrificed mice, as described under “Experimental Procedures.” *B*, hippocampal proteins from control (left panel) and stressed mice (right panel) were separated on 2D-PAGE, detected with silver staining. Representative images are shown. Sixty-three differentially changed protein spots identified based on reproducible triplicated samples are indicated with arrow. Twenty-one spots decreased (left), whereas 42 spots increased (right) after chronic restraint stress.

Recently we reported that C57BL/6 mice treated with restraint daily 2 h for 14 days exhibited depressive-like behaviors in the tail suspension test and forced swim test, and these behavioral changes lasted for more than 3 months (6, 7, 27). In line with the same stress treatment paradigm, male C57BL/6 mice were exposed to restraint for 2 h daily for 9 or 14 days as described under Experimental Procedures. Unstressed animals were used as controls. The experimental design is presented in Fig. 1*A*. Whole hippocampi were isolated on the last day of stress treatment and hippocampal proteins from each individual were separated by 2D-PAGE based on their size and pI value. Hippocampal protein expression profiles of control, 9-day- and 14-day-chronic restraint stress mice were compared (Fig. 1*B* and supplemental Fig. S1). We found, in triplicated samples, that the expression of sixty-three proteins changed by more than 1.3-fold in the stressed mouse; 21 proteins decreased (left, control mice) and those of 42 increased (right, stressed mice) following exposure to stress (Fig. 1*B*).

**Fig. 2.**
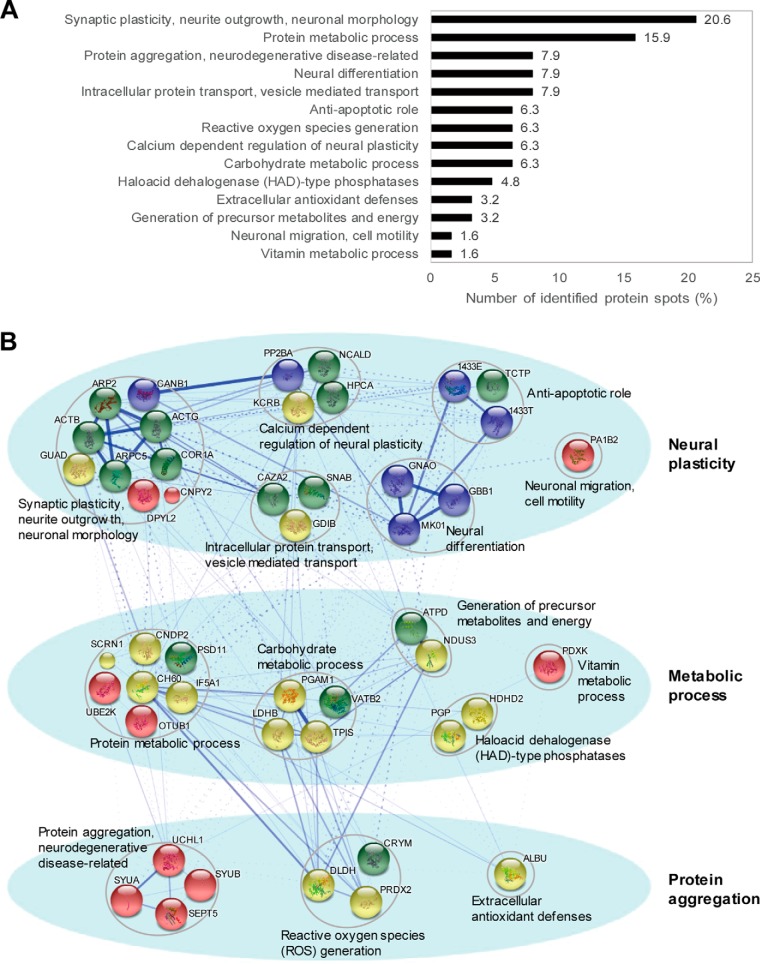
**Differentially changed proteins were identified and classified according to their biological functions.** Functional analysis based on text-mining approach (*A*) and STRING protein network analysis (*B*) showed that these identified proteins were functionally related to neural plasticity, metabolic process and protein aggregation. The thicker edge between two nodes indicates the higher confidence and dashed-lines are intercluster connections.

Each of 63 protein spots was subjected for protein identification by peptide sequencing using nanoUPLC-ESI-q-TOF tandem mass spectrometry (Table I). Upon further analysis based on their known biological functions using text-mining approach (Table I and Fig. 2*A*) and protein interaction network analysis (STRING version 10.0; http://string-db.org/) with additional clustering methods (Fig. 2*B*), we found that proteins functioning in synaptic plasticity, neurite outgrowth and neuronal morphology (20.6%) were the most enriched, followed by proteins related to protein metabolic process (15.9%), protein aggregation, neurodegenerative diseases (7.9%), neural differentiation (7.9%), intracellular protein transport, vesicle mediated transport (7.9%), antiapoptotic role (6.3%), reactive oxygen species generation (6.3%), calcium-dependent regulation of neural plasticity (6.3%), carbohydrate metabolic processes (6.3%), haloacid dehalogenase (HAD)-type phosphatases (4.8%), extracellular antioxidant defenses (3.2%), generation of precursor metabolites and energy (3.2%), neuronal migration, cell motility (1.6%), and vitamin metabolic processes (1.6%). As shown in Fig. 2*B*, these identified proteins could be classified with respect to three major functions; neural plasticity, metabolic process, and protein aggregation, which is novel resource for molecular understanding of depression.

**Table I:** Proteins differentially changed because of chronic restraint stress in the chronic restraint stress (CRS) mouse model, as revealed by proteomic studies Each of 63 protein spots from Fig. 1*B* was identified using nanoUPLC-ESI-q-TOF tandem mass spectrometry, classified based on their biological process.

Spot #	Accession #	Uniprot ID	Protein name	pl	Mass	Mascot Score	# of Distinct Peptides	% Coverage	Ratio (CRS/Control)		*p* value
									9D	14D	
**Protein metabolic process**											
1	Q9CZC8	SCRN1	Secernin-1	4.67	46297	334	16	36	−2.00	−2.21	5.000E-03
2	Q9D1A2	CNDP2	Cytosolic non-specific dipeptidase	5.43	52734	582	26	52	−1.59	1.21	1.915E-04
3	Q9D1A2	CNDP2	Cytosolic non-specific dipeptidase	5.43	52734	469	13	32	−2.52	1.21	1.000E-03
30	Q9D1A2	CNDP2	Cytosolic non-specific dipeptidase	5.43	52734	494	21	56	1.38	1.46	3.700E-02
19	P63242	IF5A1	Eukaryotic translation initiation factor 5A-1	5.07	16821	40	2	22	−2.81	−2.50	3.903E-05
63	P63242	IF5A1	Eukaryotic translation initiation factor 5A-1	5.07	16821	64	3	30	4.70	3.11	7.108E-07
24	P63038	CH60	60 kDa heat shock protein, mitochondrial	5.91	60917	17	3	4	1.92	1.32	4.141E-06
34	Q8BG32	PSD11	26S proteasome non-ATPase regulatory subunit 11	6.08	47407	593	23	47	1.31	1.21	6.581E-05
43	Q7TQI3	OTUB1	Ubiquitin thioesterase OTUB1	4.85	31250	127	8	38	1.31	1.79	2.108E-04
57	P61087	UBE2K	Ubiquitin-conjugating enzyme E2 K	5.33	22393	121	5	32	1.49	1.28	2.100E-02
**Carbohydrate metabolic process**											
29	P62814	VATB2	V-type proton ATPase subunit B, brain isoform	5.57	56515	1069	44	72	3.99	4.70	2.000E-03
45	P16125	LDHB	l-lactate dehydrogenase B chain	5.7	36549	432	18	41	2.31	2.51	1.718E-05
53	Q9DBJ1	PGAM1	Phosphoglycerate mutase 1	6.67	28814	427	16	68	1.32	1.29	1.454E-05
56	P17751	TPIS	Triosephosphate isomerase	5.56	32171	276	13	45	2.11	2.33	3.024E-04
**Vitamin metabolic process**											
44	Q8K183	PDXK	Pyridoxal kinase	5.88	34993	488	22	49	4.21	3.02	1.222E-05
**Generation of precursor metabolites and energy**											
21	Q9D3D9	ATPD	ATP synthase subunit delta, mitochondrial	4.98	17589	28	2	32	−1.19	−1.32	1.200E-02
55	Q9DCT2	NDUS3	NADH dehydrogenase [ubiquinone] iron-sulfur protein 3, mitochondrial	6.67	30131	332	11	44	2.01	1.89	5.183E-05
**Haloacid dehalogenase (HAD)-type phosphatases**											
9	Q3UGR5	HDHD2	Haloacid dehalogenase-like hydrolase domain-containing protein 2	5.7	28730	120	13	61	−2.39	−3.21	3.528E-05
52	Q3UGR5	HDHD2	Haloacid dehalogenase-like hydrolase domain-containing protein 2	5.7	28730	164	15	52	3.11	3.30	6.398E-09
46	Q8CHP8	PGP	Phosphoglycolate phosphatase	5.2	34519	222	7	33	1.80	−1.57	3.372E-04
**Intracellular protein transport, vesicle mediated transport**											
7	P47754	CAZA2	F-actin-capping protein subunit alpha-2	5.57	32947	352	15	50	−1.92	−2.21	5.197E-06
48	P47754	CAZA2	F-actin-capping protein subunit alpha-2	5.57	32947	513	21	58	2.71	3.33	1.339E-07
31	Q61598	GDIB	Rab GDP dissociation inhibitor beta	5.93	50505	426	25	50	1.99	1.31	5.000E-03
32	Q61598	GDIB	Rab GDP dissociation inhibitor beta	5.93	50505	1336	65	70	2.71	1.70	3.291E-05
49	P28663	SNAB	Beta-soluble NSF attachment protein	5.32	33535	647	23	60	1.43	1.23	3.920E-05
**Neuronal migration, cell motility**											
12	Q61206	PA1B2	Platelet-activating factor acetylhydrolase IB subunit beta	5.57	25565	244	10	35	−1.41	−1.09	7.353E-04
**Neural differentiation**											
5	P18872	GNAO	Guanine nucleotide-binding protein G(o) subunit alpha	5.34	40059	616	28	36	−3.21	−1.79	6.652E-06
6	P18872	GNAO	Guanine nucleotide-binding protein G(o) subunit alpha	5.34	40059	402	22	49	−3.41	−1.23	6.070E-04
41	P18872	GNAO	Guanine nucleotide-binding protein G(o) subunit alpha	5.34	40059	631	23	43	2.88	2.01	8.003E-05
42	P63085	MK01	Mitogen-activated protein kinase 1	6.5	41249	363	18	44	2.22	2.49	1.507E-05
47	P62874	GBB1	Guanine nucleotide-binding protein G(I)/G(S)/G(T) subunit beta-1	5.6	37353	570	24	55	1.72	1.98	8.760E-07
**Synaptic plasticity, neurite outgrowth, neuronal morphology**											
18	Q9CPW4	ARPC5	Actin-related protein 2/3 complex subunit 5	5.47	16278	271	9	37	−2.70	−2.72	1.395E-04
62	Q9CPW4	ARPC5	Actin-related protein 2/3 complex subunit 5	5.47	16278	235	8	26	2.79	2.11	3.627E-04
20	Q63810	CANB1	Calcineurin subunit B type 1	4.64	19288	136	13	65	−1.49	−1.10	1.300E-02
25	O08553	DPYL2	Dihydropyrimidinase-related protein 2 (CRMP2)	5.95	62239	47	4	4	2.35	4.02	9.666E-07
26	O08553	DPYL2	Dihydropyrimidinase-related protein 2 (CRMP2)	5.95	62239	57	2	2	1.70	2.41	8.359E-07
27	O89053	COR1A	Coronin-1A	6.05	50957	564	22	52	3.22	2.28	4.698E-06
33	Q9R111	GUAD	Guanine deaminase	5.36	50981	711	27	61	1.77	1.29	2.445E-06
35	P60710	ACTB	Actin, cytoplasmic 1	5.29	41710	1160	38	67	2.83	2.41	4.293E-08
37	P60710	ACTB	Actin, cytoplasmic 1	5.29	41710	1787	64	71	3.12	2.77	2.218E-04
36	P63260	ACTG	Actin, cytoplasmic 2	5.31	41766	1795	70	73	2.77	2.50	4.822E-05
39	P61161	ARP2	Actin-related protein 2	6.29	44732	204	8	21	2.22	2.59	3.618E-07
40	P61161	ARP2	Actin-related protein 2	6.29	44732	486	17	32	1.89	1.47	4.540E-02
61	Q9QXT0	CNPY2	Protein canopy homolog 2	4.95	20754	46	2	16	1.81	1.55	4.251E-04
**Calcium dependent regulation of neural plasticity**											
4	Q04447	KCRB	Creatine kinase B-type	5.4	42686	1645	58	70	−4.21	1.02	1.118E-04
17	P84075	HPCA	Neuron-specific calcium-binding protein hippocalcin	4.87	22413	194	15	55	−1.51	−1.30	1.861E-04
51	P63328	PP2BA	Serine/threonine-protein phosphatase 2B catalytic subunit alpha isoform	5.58	58606	381	17	37	2.81	2.71	7.667E-04
60	Q91X97	NCALD	Neurocalcin-delta	5.23	22231	193	7	24	1.77	−1.02	3.500E-02
**Protein aggregation, neurodegenerative disease-related**											
11	Q9R0P9	UCHL1	Ubiquitin carboxyl-terminal hydrolase isozyme L1	5.14	24822	280	11	30	−2.02	−1.75	2.970E-02
54	Q9R0P9	UCHL1	Ubiquitin carboxyl-terminal hydrolase isozyme L1	5.14	24822	384	14	43	3.63	1.76	1.104E-06
15	Q91ZZ3	SYUB	Beta-synuclein	4.38	14043	141	3	29	−1.91	−1.42	3.148E-07
16	O55042	SYUA	Alpha-synuclein	4.74	14476	284	10	44	−1.19	−1.47	2.695E-04
38	Q9Z2Q6	SEPT5	Septin-5	6.21	42721	526	16	27	1.32	1.30	4.286E-06
**Reactive oxygen species generation**											
8	O54983	CRYM	Ketimine reductase mu-crystallin	5.44	33502	869	31	54	−1.30	−1.19	1.260E-05
14	Q61171	PRDX2	Peroxiredoxin-2	5.2	21765	98	7	18	−2.70	−4.21	9.768E-07
59	Q61171	PRDX2	Peroxiredoxin-2	5.2	21765	658	28	66	1.69	1.88	1.859E-04
28	O08749	DLDH	Dihydrolipoyl dehydrogenase, mitochondrial	7.99	54238	542	25	46	3.69	1.19	2.300E-04
**Extracellular antioxidant defenses**											
22	P07724	ALBU	Serum albumin	5.75	68648	187	7	9	2.71	1.99	1.532E-08
23	P07724	ALBU	Serum albumin	5.75	68648	105	5	9	1.79	1.42	1.966E-06
**Anti-apoptotic role**											
10	P68254	1433T	14-3-3 protein theta	4.69	27761	531	31	62	−2.53	−2.33	1.348E-07
13	P63028	TCTP	Translationally-controlled tumor protein	4.76	19450	337	14	37	−1.21	−1.42	1.638E-04
58	P63028	TCTP	Translationally-controlled tumor protein	4.76	19450	166	6	30	2.12	1.59	2.000E-03
50	P62259	1433E	14-3-3 protein epsilon	4.63	29155	1176	40	61	1.72	1.61	3.598E-04

##### Kinetics of Protein Profile Changes in Response to Chronic Restraint Stress

**Fig. 3.**
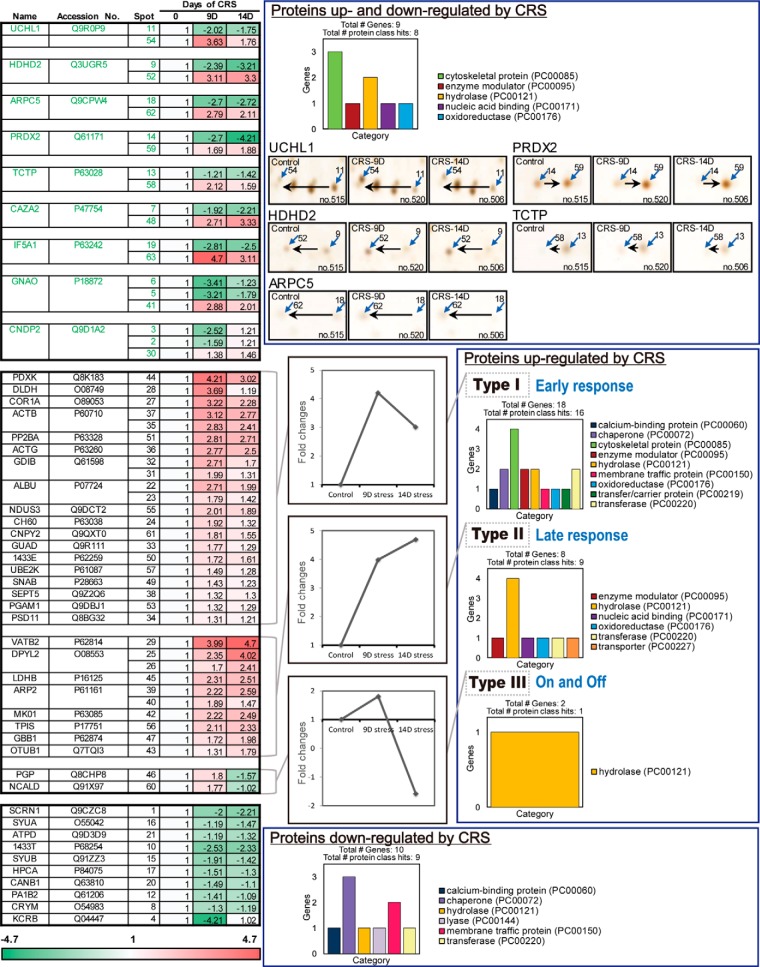
**Expression kinetics patterns of CRS-regulated proteins and protein class analysis using PANTHER.** Kinetics of spot intensity changes (normalized to control) by CRS were shown in the heat map. Spot intensity was measured using auto-processing 2-DE analysis software SameSpots. Proteins up- and down-regulated by CRS showed apparent spot shift induced by post-translational modifications. PANTHER protein class analysis showed that proteins up-regulated by CRS were mainly consisted of cytoskeletal protein and hydrolase. Most proteins down-regulated by CRS were chaperone and membrane traffic protein.

We further classified the identified proteins into three groups on the basis of the kinetics of expression changes as shown in heat map (Fig. 3). The first group consisted of the proteins both up- and down-regulated by chronic restraint stress, which presumably represent proteins having different post-translational modifications that shifted on 2D-PAGE by pI. UCHL1, HDHD2, ARPC5, PRDX2, TCTP, CAZA2, IF5A1, GNAO and CNDP2 were highlighted in green in the heat map, because these spots of UCHL1, HDHD2, ARPC5, TCTP, CAZA2, GNAO, and CNDP2 shifted to lower pI regions (acidic shift) by chronic restraint stress, whereas the spots of PRDX2 and IF5A1 shifted to higher pI regions (basic shift). Representative spot shifts are shown in the right panel. Protein class analysis using PANTHER classification system (PANTHER™ Protein Class version 11.1; http://pantherdb.org/) revealed that these proteins are related to cytoskeletal protein and hydrolase.

The second group includes proteins that were up-regulated by cumulative restraint stress, and they were then divided into three subgroups (Type I to III). A group of 18 proteins including PDXK and DLDH (Type I) peaked on day 9 and slightly decreased thereafter. Therefore, we classified these proteins as “early response to stress” proteins. Another group of 8 proteins including VATB2 and DPYL2 (Type II) was classified as “late response” proteins, which showed gradual increases up to day 14. The third group including PGP and NCALD (Type III) was called the 'on and off' response proteins because the kinetic changes of these proteins showed an increase on day 9, but a sharp decrease on day 14. As shown in protein class analysis data, proteins in this group mainly consisted of cytoskeletal protein (Type I) and hydrolase (Type II and III).

The last group contains ten proteins downregulated by stress, including KCRB and 1433T. They were at minimum expressions on day 9 or day 14. Most of these proteins are chaperone and membrane traffic proteins, implying that chronic restraint stress decreases chaperone activity. These results suggest that the amount and expression kinetics of the stress-regulated proteins were dynamically regulated by the length of stress treatment.

##### UCH-L1 Is the Protein Most Changed by Chronic Restraint Stress

**Fig. 4.**
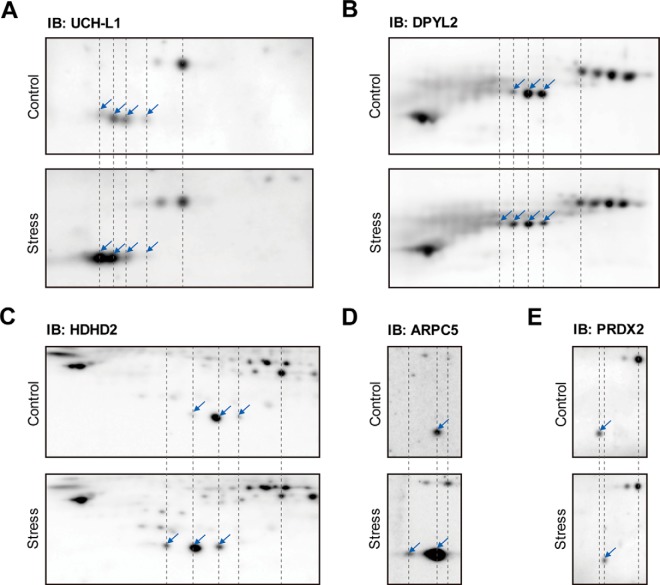
**Identification of differentially changed proteins confirmed by Western blot analysis.** Expression patterns of each protein in control and stressed mouse hippocampus were compared as shown. Location of protein spots were marked with arrows. *A*, UCH-L1 (Ubiquitin C-terminal hydrolase isozyme L1), *B*, DPYL2 (Dihydropyrimidinase-related protein 2), *C*, HDHD2 (Haloacid dehalogenase-like hydrolase domain-containing protein 2), *D*, ARPC5 (Actin-related protein 2/3 complex subunit 5) and *E*, PRDX2 (Peroxiredoxin-2) were detected on 2D-PAGE.

To validate the proteins identified by proteomic analysis, hippocampal tissue lysates from control and stressed mice were examined by Western blot analysis on 2D-PAGE using specific antibodies (Fig. 4). Mini-gel 2D-PAGE was performed for Western blot analysis which has better detection sensitivity than silver staining; therefore, the spots could be detected with the smaller amounts of proteins. We could also examine the overall profile of each identified protein in a single gel without missing the spots by employing mini-2D-Western blot analysis.

Western blot analysis with anti-UCH-L1 confirmed that UCH-L1 spots shifted to the left acidic region in stressed mice along with increases in amount, showing the most significant changes (Fig. 4*A*). Western blot analysis indicated that the spots of DPYL2 (Fig. 4*B*), HDHD2 (Fig. 4*C*), and ARPC5 (Fig. 4*D*) also moved to the acidic direction in chronically stressed samples. Using 2D-western, several populations of each protein were identified in addition to those found in silver stained gels (UCH-L1, DPYL2 and HDHD2). The stress blot of ARPC5 (Fig. 4*D*, lower panel) was obtained by high exposure to show the moved spot (acidic spot) more clearly, so the spot in the control position (basic spot) seemed to have a higher intensity than in control blot (upper panel). This difference was confirmed by the intensities of standard spots. Overall, these spot shifts examined by Western blots were consistent with the results seen on 2D-PAGE (Fig. 1*B*). Acidic protein spot shift to lower pI values could happen when Cys oxidation to sulfinic and sulfonic acid, phosphorylation at Ser, Thr and Tyr, or acetylation at Lys occurs. Employing MS analysis of UCH-L1, we concluded that the Cys oxidations were the major response induced by chronic stress (Table II). These results are consistent with the previous report that chronic restraint stress increases oxidative stress in the hippocampus (7). Western blot analysis confirmed that PRDX2 spots moved to the right (basic) direction (Fig. 4*E*), as the result on silver stained spots on 2D-PAGE (Fig. 1*B*). Because oxidized PRDX2 is readily removed (21), these spots likely represent newly synthesized PRDX2 as described in the previous study (21). These results suggest that oxidized proteins accumulate in the hippocampus during chronic restraint stress.

**Table II:** Cysteine oxidative modifications of UCH-L1 in the hippocampus of control and stress-treated mice Peptides with the highest ions score for each spot were tabulated. Modified residues were highlighted in **bold** and *italic*. ND, modified peptides were not detected. MS/MS spectra of modified peptides are presented in supplementary Fig. S7.

Residue	Modification	Start-End	Mass (*m/z*) Experimental	Mass Theoretical	Delta Mass (Da)	Score							Sequence
						Control			Stress				
						spot 3	spot 2	spot 1	spot 4	spot 3	spot 2	spot 1	
C90	Dioxidation	84–115	851.6668	3402.6725	−0.0344	54	109	ND	61	104	ND	ND	QTIGNS***C***GTIGLIHAVANNQDKLEFEDGSVLK
	Trioxidation		1140.5627	3418.6674	−0.0012	156	163	59	127	156	151	ND	
	Cys-SO_2_-SH		1145.8849	3434.6446	−0.0117	103	61	15	106	126	86	ND	
C152	Trioxidation	136–153	987.9305	1973.8497	−0.0033	71	36	23	69	68	42	ND	NEAIQAAHDSVAQEGQ***C***R
	Cys-SO_2_-SH		995.9210	1989.8269	0.0006	74	55	ND	64	46	ND	ND	

Because TPIS, PGAM1, and PDXK identified in Fig. 3, have also been reported as important glycolysis enzymes altered in schizophrenia (28, 29) and similarities between psychiatric disorders have been reported in recent studies (30, 31), we investigated total expression level of these proteins employing 1D-Western blot analysis (supplemental Fig. S2*A*). GAPDH was used as loading control because ACTB and ACTG increased almost 3-fold in the CRS mouse (Table I). Only PDXK showed significant increase (1.2-fold increase in 9-day stress, 1.39-fold increase in 14-day stress) by chronic restraint stress, whereas total changes in TPIS and PGAM1 were not significant because these proteins may have several populations (supplemental Fig. S2*B*). Increased PDXK may cause change in vitamin B6 level which is associated with various enzymatic pathways, however, these changes were not dramatic as the UCH-L1 showed.

##### Chronic Restraint Stress Induces Post-translational Modifications of UCH-L1

**Fig. 5.**
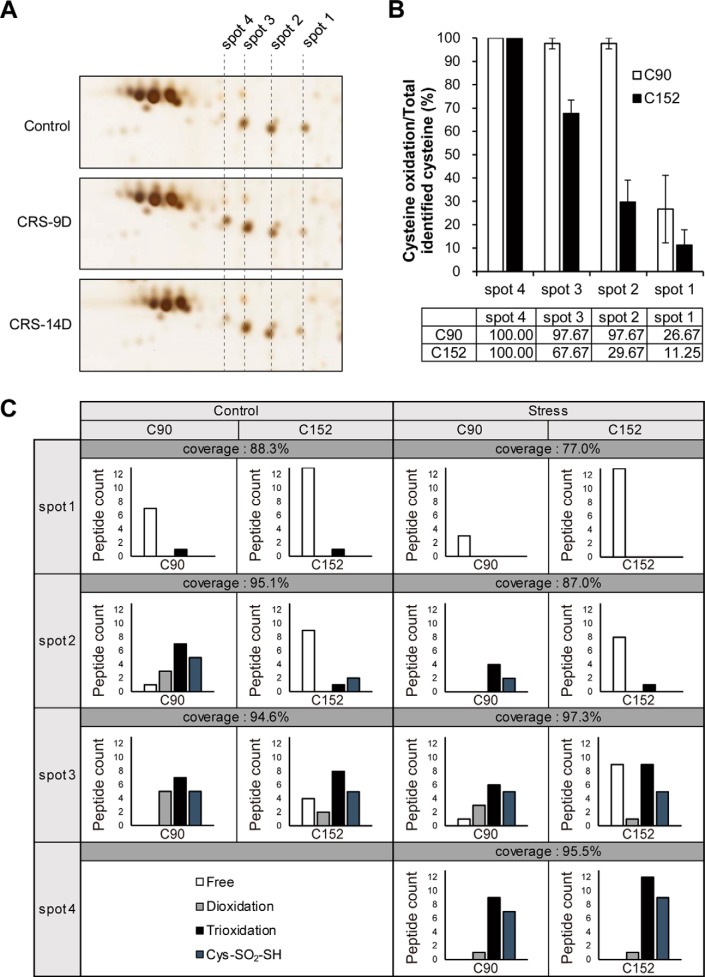
**Oxidation of Cys152 is a main cause of UCH-L1 spot shift by CRS.** *A*, Four populations of UCH-L1 were found and re-numbered as shown. *B*, Oxidations at Cys90 and Cys152 of UCH-L1 were examined by MS/MS analysis employing SEMSA strategy and MOD^i^ database. Cys152 of UCH-L1 was gradually oxidized from spot 1 to spot 4 whereas active site Cys90 is more sensitive to oxidative modifications. *C*, The number of modified peptides of Cys90 and Cys152 of UCH-L1 spots were presented. Various oxidative modifications such as dioxidation, trioxidation and conversion of Cys to thiosulfonic acid (Δm = +64 Da) were found.

Extensive analysis of UCH-L1 spot shifts (Fig. 4*A*) led us to identify at least four major UCH-L1 spots, which were re-numbered and presented in Fig. 5*A*. Spots 1 (spot 11 in Fig. 1*B*) and 2 decreased on day 9 of stress treatment, whereas spots 3 and 4 (spot 54 in Fig. 1*B*) increased. On day 14 of stress treatment, these changes were partially reversed, whereas the total intensity of the four spots remained unchanged.

To confirm that the molecular changes in UCH-L1 were responsible for UCH-L1 spot shifts because of restraint stress, we carried out a comprehensive PTM analysis for each of UCH-L1 spots. PTMs of spots 1 to 4 in the hippocampus of control and stressed mice were analyzed by peptide sequencing with nanoUPLC-ESI-q-TOF-MS/MS analysis, using SEMSA strategy which allowed for sensitive detection of low abundant PTMs (22) and MOD^i^ (23) and Mascot algorithms for searching the unknown PTMs.

Table II and supplemental Table S1 summarize the results of this PTM analysis of UCH-L1. These results suggest that spot shifts of UCH-L1 to the acidic region are caused by distinct Cys oxidations. Various Cys oxidative modifications including dioxidation, trioxidation, and conversion of Cys to thiosulfonic acid (Cys-SO_2_-SH, Δm = +64 Da) (32) occurred at Cys90 and Cys152. The Cys90, active site of UCH-L1, was fully oxidized in spots 2, 3 and 4. In contrast, Cys152 was increasingly oxidized from spot 1 to spot 4 (Fig. 5*B*), which is a major cause of spot shift. Dioxidation at Cys152, presented in supplemental Table S1, was classified as ambiguous PTM because the accurate spectra for that modification could not be annotated, but because Cys152 is the only Cys in that peptide (^136^NEAIQAAHDSVAQEGQ*C*R^153^) we included the modification for the calculation in this case. Spot 1 from stressed sample had no identified cysteine modifications, probably because of the low abundance of spot 1, although we analyzed the PTMs of several pooled spots (stress spot 1). Other PTMs of UCH-L1 are summarized in Table III where representative tryptic peptides are shown and the modified residues are highlighted in red. Phosphorylations were detected at Thr85, Ser119 and Ser188. Acetylations at Lys71 and Lys123 occurred. Phosphorylation at Thr85 was detected concurrently with trioxidation at Cys90. Phosphorylation at Ser119 was detected in all spots and phosphorylation at Ser188 also did except for the spot 1 in stressed sample. Acetylations at Lys71 and Lys123 were not detected in spot 1 of control and stressed mice.

**Table III:** Post-translational modifications of UCH-L1 in the hippocampus of control and stress-treated mice Peptides with the highest ions score for each spot were tabulated. Modified residues were highlighted in **bold** and *italic*. ND: Modified peptides were not detected, N, Q: Deamidated, deamidated; M, oxidation. MS/MS spectra of modified peptides are presented in supplementary Fig. S7.

Modification	Residue	Start-End	Mass (*m*/*z*) Experimental	Mass Theoretical	Delta Mass (Da)	Score (total mascot score)							Sequence
						Control			Stress				
						spot 3 (923)	spot 2 (1241)	spot 1 (694)	spot 4 (505)	spot 3 (1055)	spot 2 (658)	spot 1 (394)	
Phosphorylation	T85/C90_trioxi	84–115	1167.2183	3498.6338	−0.0007	42	162	ND	156	112	ND	ND	Q***T***IGNS***C***GTIGLIHAVANNQDKLEFEDGSVLK
	S119	116–129	879.8976	1757.7873	−0.0066	77	63	73	76	68	70	75	QFL***S***ETEKLSPEDR
	S188	179–199	1155.9977	2309.9875	−0.0067	68	58	62	58	62	58	ND	MPFPVNHGA***S***SEDSLLQDAAK
Acetylation	K71	66–78	764.4047	1526.7828	0.0121	42	32	ND	25	41	31	ND	QIEEL***K***GQEVSPK
	K123	116–129	574.2860	1719.8315	0.0047	30	16	ND	16	11	ND	ND	QFLSETE***K***LSPEDR

We searched for additional modifications causing more acidic spots of UCH-L1 other than Cys oxidative modifications. Phosphorylation at Thr85 was only detected when trioxidation at Cys90 was detected (Table III) and trioxidation at Cys90 was detected in all spots (except the spot 1 in stressed mice in which no Cys modifications were found, Table II), so it could be suggested that phosphorylation at Thr85 can be the modification to cause the acidic shift from spot 1 to other spots (2, 3, and 4). However, there are other possible interpretations of these shifts because it is not possible to identify 100% of PTMs with MS analysis. This is true also for the phosphorylation at Ser145, which was identified in all spots regardless of the oxidation of Cys152 (supplemental Table S1). Generally, phosphorylation was equally well detected in all spots (Table III and supplemental Table S1). Acetylations at Lys71 and Lys123 were not found in spot 1 (Table III), so it can be a criterion that distinguishes spot 1 from other spots. Nevertheless, we were able to reaffirm that it is Cys152 oxidation that can explain the change over all spots.

Fig. 5*C* presents the peptide counts of various Cys oxidative modifications for each spot. This analysis suggests that the degree of oxidation at Cys152 residue was a major cause of UCH-L1 spot shifts, also consistent with the previous report that Cys90 and Cys152 of UCH-L1 have different redox-sensitivity (16). The present study also confirmed that the differences between various populations of a single protein show acidic shift by the oxidative stress. Intriguingly, because only a small amount of UCH-L1 is preserved its active site Cys90 in an active and unmodified state, UCH-L1 seems to play a critical role in response to oxidative stress in addition to its original enzymatic activity.

##### UCH-L1 Is Expressed in Hippocampal Neuronal Cells

**Fig. 6.**
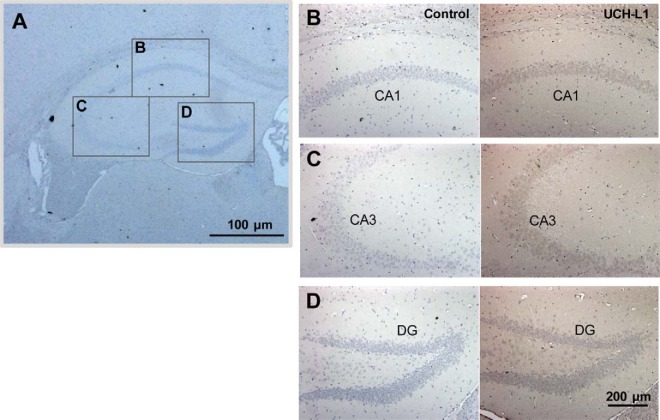
**UCH-L1 is highly expressed in hippocampal neuronal cells.** Mouse brain tissue sections were subjected to immunohistochemistry using anti-UCH-L1 antibody. Photomicrographs showing the expression of UCH-L1 (*B–D*) in the hippocampus of mice. UCH-L1 is mainly distributed in pyramidal neurons in the CA1 (*B*), and CA3 (*C*), and granule cells in the dentate gyrus (*D*) (right panel). Left panel is negative control. Approximate locations for the CA1, CA3, and DG regions are marked by box (*A*).

To determine in which type of cells in hippocampus UCH-L1 exerts its activity, we examined the distribution of UCH-L1 in mouse hippocampal tissue (Fig. 6). Immunohistochemistry was carried out using UCH-L1 antibody coupled with DAB staining, and cell nuclei were stained using hematoxylin. Approximate locations for the CA1, CA3, and DG regions are marked in Fig. 6*A*. UCH-L1 was distributed mainly in neuronal cells throughout the brain (Fig. 6*B*–6*D*). In the hippocampus, UCH-L1 (right panel) was expressed in pyramidal cells of the CA1 (Fig. 6*B*) and CA3 (Fig. 6*C*) and in granule cells in the dentate gyrus (Fig. 6*D*). Negative controls are in left panels. UCH-L1 appeared to be also expressed in microglia and astrocytes.

##### UCH-L1 Protects HT-22 Cells Against Oxidative Stress

**Fig. 7.**
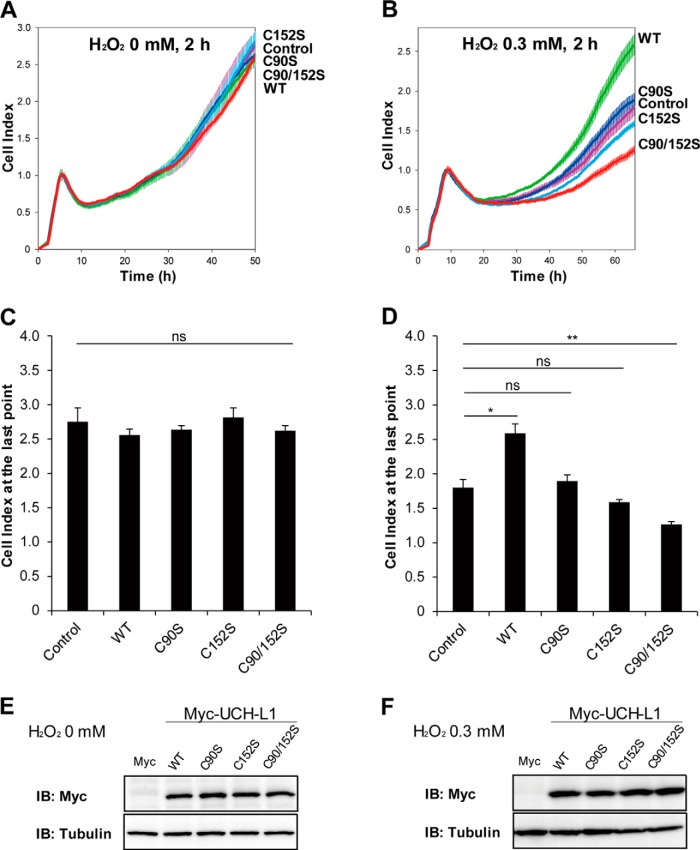
**UCH-L1 protects HT-22 cells from H_2_O_2_ oxidative stress.** *A* and *B,* HT-22 cells were transfected with empty pcDNA3.1/myc-His(-) A vector (designated as Control) and the same plasmid carrying UCH-L1 WT, C90S, C152S and C90/152S mutant constructs (WT, C90S, C152S and C90/152S, respectively). Cells were treated with 0 or 0.3 mm H_2_O_2_ in HBSS for 2 h before transferred to RTCA 96-well plate (1 × 10^4^ cells/well) for further analysis. Cell survivals were monitored by xCELLigence real-time cell analysis instrument. *C* and *D*, There were no significant differences between 0 mm H_2_O_2_ treated cells, but wild-type UCH-L1 effectively protected HT-22 cells from 0.3 mm H_2_O_2_. Cells overexpressing C90/152S mutant were even more vulnerable to oxidative stress. *E* and *F*, Overexpressed UCH-L1 with C-terminal myc tag in each experiment was verified by Western blot analysis. Data were shown as the means ± S.E. of triplicates (ns: not significant, *p < 0.05, **p < 0.01).

Because the kinetics of oxidation of UCH-L1 at Cys90 and Cys152 following chronic restraint stress are different (Fig. 5), we investigated whether different Cys residues of UCH-L1 had specific roles in response to oxidative stress. We examined the effects of various Cys mutants of UCH-L1 on the survival of HT-22 mouse hippocampal neuronal cells subjected to oxidative stress. HT-22 cells transiently transfected with UCH-L1 wild-type (WT), and Cys mutants C90S, C152S, and C90/152S respectively, were exposed to 0 or 0.3 mm H_2_O_2_ for 2 h, and real-time cell growth patterns were monitored employing xCELLigence RTCA apparatus (Fig. 7). As shown in Figs. 7*B* and 7*D*, HT-22 cells overexpressing UCH-L1 WT exerted protective effect against 0.3 mm H_2_O_2_-induced oxidative stress, whereas no discernible differences were observed in untreated cells (0 mm H_2_O_2_, Fig. 7*A* and Fig. 7*C*). This protective effect was abolished in HT-22 cells overexpressing C90S, C152S, and C90/152S mutants. HT-22 cells overexpressing C152S and C90/152S mutants were more sensitive to oxidative stress than control cells, indicating that Cys152 plays a key role in mitigating oxidative stress. We reported previously (16), that Cys90 is in the active site and is readily oxidized, and that Cys152 is also a redox sensitive residue to be oxidized by oxidative stress. Expression levels of each type of UCH-L1 were confirmed by Western blotting (Fig. 7*E* and Fig. 7*F*). Because endogenous level of UCH-L1 in HT-22 cells is low compared with the overexpressed UCH-L1 (supplemental Fig. S3), these protective effects on cell viability are mainly caused by overexpression of UCH-L1. These results suggest that redox sensitive Cys residues in UCH-L1 protect the hippocampal cells from oxidative damage.

To investigate UCH-L1 Cys modifications in HT-22 cells as well as in the tissue, HT-22 cells overexpressing Myc-tagged wild-type UCH-L1 were treated with 0 or 1 mm of H_2_O_2_ for 1 h at 37 °C and cellular proteins were subjected to 2D-PAGE for MS analysis (supplemental Fig. S4). Stoichiometry changes to oxidized spots of overexpressed UCH-L1 in HT-22 cells were lower than those of tissue ones (supplemental Fig. S4*A* and S4*B*). These low abundant oxidized spots can be identified as UCH-L1 with MS/MS, however, were not enough for PTM analysis. In our experience, PTMs of a protein are well identified with higher stoichiometry in tissues than in cultured cells, and PTMs of endogenous proteins are much better identified than transiently overexpressed protein in cells. supplemental Fig. S5 shows overall protein profiles of HT-22 cells (full images of 2D-gels presented in supplemental Fig. S4), indicating the very low expression of endogenous UCH-L1 in this cell line.

**Fig. 8.**
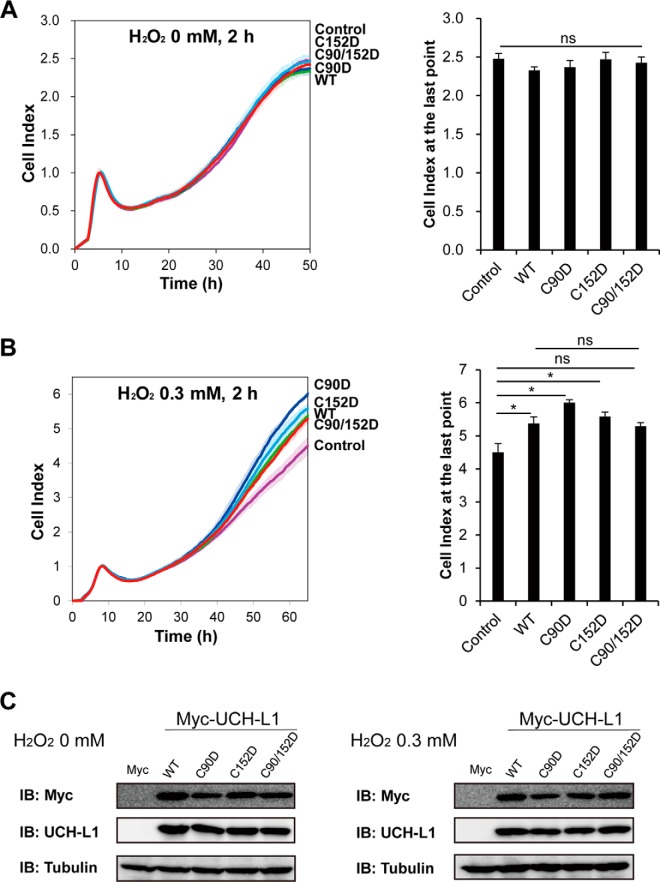
**HT-22 cells overexpressing C90D and C152D mutants of UCH-L1 have a protective effect comparable to that of wild-type UCH-L1 in response to H_2_O_2_ oxidative stress.** HT-22 cells were transfected with empty pcDNA3.1/myc-His(-) A vector (Control) and the same plasmid carrying UCH-L1 WT, C90D, C152D and C90/152D mutant constructs. Cells were treated with 0 (*A*) or 0.3 mm (*B*) H_2_O_2_ in HBSS for 2 h, transferred to RTCA 96-well plate at the density of 1 × 10^4^ cells/well and cell survivals were monitored by xCELLigence real-time cell analysis instrument. No discernible differences were detected between cells treated with 0 mm H_2_O_2_ (*A*), whereas cells overexpressing WT, C90D and C152D UCH-L1 showed protective effect against 0.3 mm H_2_O_2_ (*B*). Cells overexpressing C90/152D UCH-L1 also showed increase in cell viability but the difference was not statistically significant. *C*, Overexpressed UCH-L1 with C-terminal Myc-tag in each experiment was verified by Western blot analysis. Data were shown as the means ± S.E. of triplicates (ns: not significant, *p < 0.05).

To further investigate whether the protective function of UCH-L1 is related to the loss of its enzyme function caused by oxidative modifications of its Cys residues, we examined the protective effects of various Cys to Asp mutants (C90D, C152D, and C90/152D) of UCH-L1, which mimic oxidized Cys residues. We determined the cell viabilities of HT-22 cells overexpressing WT and C90D, C152D and C90/152D of UCH-L1 subjected to oxidative stress, employing xCELLigence RTCA. Overexpression of Cys to Asp mutants of UCH-L1 had no effect on cell viability without treatment of oxidative stress (Fig. 8*A*). Intriguingly, these Cys to Asp mutants showed protective effect comparable to that of wild-type UCH-L1 in response to oxidative stress (0.3 mm H_2_O_2_) (Fig. 8*B*). Cells overexpressing C90/152D mutant also showed some increase in cell viability when treated with 0.3 mm H_2_O_2_, but the difference was not statistically significant. Overexpression of each UCH-L1 mutant was verified by Western blot analysis (Fig. 8*C*). This study demonstrated that the protective effect of wild-type UCH-L1 was not from massive overexpression of protein containing reactive thiols. Cys to Asp mutants of UCH-L1 seemed to mimic Cys oxidized wild-type UCH-L1, which suggests that oxidized UCH-L1 itself is exerting the neuroprotective function.

To investigate whether oxidized form of UCH-L1 is causing its precipitation, we fractionated HT-22 cells overexpressing Cys mutants (both Cys to Ser and Cys to Asp mutants) treated with 0 or 0.3 mm H_2_O_2_ (supplemental Fig. S6). We previously reported that UCH-L1 is a highly soluble protein and that the N-terminal truncated form of UCH-L1, which serves as an ROS scavenger in cells, is relatively easily aggregated and degraded (33). For this reason, we performed cell fractionation of soluble and insoluble components of UCH-L1 employing the mild condition used by Kabuta *et al.* (17), to obtain the 1% Triton X-100-insoluble fraction of UCH-L1. UCH-L1 levels in both soluble and insoluble fractions were confirmed by Western blot analysis (supplemental Fig. S6*A*, Cys to Ser mutants; supplemental Fig. S6*B*, Cys to Asp mutants). Because the amounts of insoluble fractions were small, they were concentrated 5-fold for comparison with soluble fractions, to obtain UCH-L1 bands for further densitometry assays (supplemental Fig. S6, bar graphs). The significant difference in protein solubility caused by H_2_O_2_ was only found in the insoluble fraction of WT UCH-L1 (supplemental Fig. S6, *A* and *B*, right bar graphs) and C152D (supplemental Fig. S6*B*, right bar graph). Specifically, Cys to Ser mutants tended to show patterns of increasing insoluble fraction after H_2_O_2_ treatment, whereas Cys to Asp mutants tended to show no changes or decreasing patterns, although these differences were not statistically significant. Wild-type UCH-L1 is readily oxidized by H_2_O_2_, consistent with the results in Fig. 7. However, the increases in insoluble fractions did not correlate with the cell viability decreases (Fig. 7). This suggests that the solubility change of UCH-L1 is not a major factor in cell viability.

**Fig. 9.**
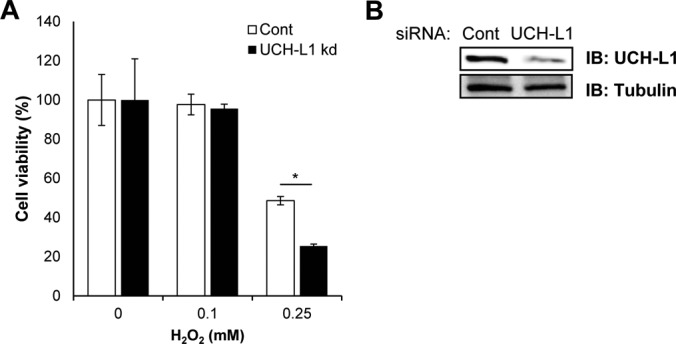
**Effect of UCH-L1 silencing in SH-SY5Y on cell viability in response to oxidative stress.** *A*, SH-SY5Y cells were transfected with control siRNA or UCH-L1 siRNA and after 72 h, cells were exposed to 0, 0.1 and 0.25 mm H_2_O_2_ for 2 h. Cell viability was measured using WST-1 reagent. SH-SH5Y cells knocking down UCH-L1 (UCH-L1 kd) have significantly decreased cell viability under stronger oxidative stress (0.25 mm H_2_O_2_). Data were shown as the means ± S.E. of triplicates (*p < 0.05). *B*, Gene silencing of UCH-L1 was confirmed by Western blot analysis.

Our results demonstrate the protective function of UCH-L1 through an overexpression model in HT-22 cells, which is a representative mouse hippocampal cell line, often used as neuronal cell model for oxidative stress studies. Because endogenous UCH-L1 in HT-22 cells is less expressed than overexpressed UCH-L1 (supplemental Fig. S3), HT-22 cells are appropriate model system to examine the gain-of-function of UCH-L1. To investigate the loss-of-function of UCH-L1, we employed SH-SY5Y human neuroblastoma cells expressing high endogenous UCH-L1. SH-SY5Y cells were transfected with the specific siRNA to knock down UCH-L1 or control siRNA for 72 h, and cell viabilities were assessed employing WST-1 cell viability assay in response to various concentrations of H_2_O_2_ (0, 0.1 and 0.25 mm) for 2 h at 37 °C (Fig. 9*A*). SH-SY5Y cells knocking down UCH-L1 showed significant decrease in cell viability when treated with oxidative stress of 0.25 mm H_2_O_2_, but not cells treated with 0 or 0.1 mm mild oxidative stress. Knockdown of UCH-L1 was verified by Western blot analysis (Fig. 9*B*). The above finding is consistent with the gain-of-function results of studies in which UCH-L1 overexpression and its oxidized form protects cells from oxidative damage (Fig. 7).

## DISCUSSION

In this study employing 2D-PAGE and MS analysis, we identified 63 proteins that changed in the hippocampus of chronic restraint stress mouse model of major depressive disorder (MDD). These proteins were classified into three groups by their function, respectively in synaptic plasticity, metabolic processes and protein aggregation. Of these 63 proteins, UCH-L1 (ubiquitin C-terminal hydrolase isozyme L1), DPYL2 (dihydropyrimidinase-related protein 2 (CRMP2)), HDHD2 (haloacid dehalogenase-like hydrolase domain-containing protein 2), ARPC5 (actin-related protein 2/3 complex subunit 5) and PRDX2 (peroxiredoxin-2) showed significant spot shifts on 2D-PAGE because of post-translational modifications (PTMs) caused by chronic stress. We demonstrated that site-specific oxidative modifications at Cys90 and Cys152 of UCH-L1 had occurred after chronic restraint stress. Overexpression of UCH-L1 in HT-22 hippocampal cells protected these cells from H_2_O_2_-caused oxidative stress, suggesting that the Cys residues, Cys90 and Cys152, of UCH-L1 play key roles in this protective effect.

We found that the first group of proteins that were significantly changed in the hippocampus of animals exposed to chronic restraint stress, ARPC5, ARP2, DPYL2, CNPY2, GUAD, SNAB, GDIB, CAZA2, COR1A, CANB1, PP2BA, KCRB, HPCA are those that regulated neural plasticity. This suggests that chronic stress induced changes in hippocampal proteins relate to synaptic plasticity. In this group, ARPC5 (actin-related protein 2/3 complex subunit 5), DPYL2 (dihydropyrimidinase-related protein 2 (CRMP2)) and CNPY2 (protein canopy homolog 2) regulate neurite outgrowth cooperatively (34–36). DPYL2 and GUAD (guanine deaminase) regulate dendrite branching and axon formation via interacting with tubulin heterodimer and promoting microtubule assembly (37–39); cytoplasmic actin ACTB (actin, cytoplasmic 1), ACTG (actin, cytoplasmic 2), transport protein SNAB (beta-soluble NSF attachment protein), GDIB (Rab GDP dissociation inhibitor beta), and CAZA2 (F-actin-capping protein subunit alpha-2) play roles in dendritic morphogenesis (40). ARP2 (actin-related protein 2) and ARPC5 form an Arp2/3 complex, nucleate actin polymerization at the dendritic spine (41); COR1A (coronin-1A) binds to Arp2/3 complex to elicit inhibitory effect (42). Calcineurin is important for synaptic plasticity and memory function (43, 44); and CANB1 (calcineurin subunit B type 1) and PP2BA (serine/threonine-protein phosphatase 2B catalytic subunit alpha isoform), are, respectively, regulatory and catalytic subunits of calcineurin, differentially regulated after chronic restraint stress. Because alteration of synaptic plasticity is important for learning and memory formation, SEPT5 (septin-5) and UCHL1 associated with cognitive function as well as ARP2 and KCRB (creatine kinase B-type) are also categorized into this group (14, 45–47). KCRB and HPCA (neuron-specific calcium-binding protein hippocalcin) decreased after chronic restraint stress, and these proteins are associated with Ca^2+^ signaling and regulation of synaptic plasticity and memory function (48).

The second group of proteins we identified as altered by chronic stress are those functioning in protein, carbohydrate, vitamin and energy-related metabolic processes (CH60, OTUB1, UBE2K, PGAM1, VATB2, LDHB, TPIS, NDUS3, IF5A1), and haloacid dehalogenase (HAD)-type phosphatases. In this group, CH60 (60 kDa heat shock protein, mitochondrial), OTUB1 (ubiquitin thioesterase OTUB1) and UBE2K (ubiquitin-conjugating enzyme E2 K), increased after chronic stress, are involved in protein metabolic process and facilitating the correct folding of protein (CH60) and ubiquitin proteasome system (OTUB1 and UBE2K). PGAM1 (phosphoglycerate mutase 1), VATB2 (V-type proton ATPase subunit B, brain isoform), LDHB (l-lactate dehydrogenase B chain) and TPIS (triosephosphate isomerase) function in carbohydrate metabolic process. NDUS3 (NADH dehydrogenase [ubiquinone] iron-sulfur protein 3, mitochondrial) is associated with energy metabolism; DLDH (dihydrolipoyl dehydrogenase, mitochondrial), classified in ROS generation group, is also involved in brain energy metabolism (49). PDXK (pyridoxal kinase) is involved in vitamin B6 metabolism and showed significant increase in CRS mouse (supplemental Fig. S2). IF5A1 (eukaryotic translation initiation factor 5A-1) and UBE2K are involved in cellular stress response (50, 51). Given that CANB1, ACTB, KCRB, PP2BA related to neural plasticity and UCHL1 also have roles in stress response (52–56), this study shows that stress-responsive proteins are well-linked to each other and metabolic changes in response to stress occur in chronic restraint stress mouse model.

The third group includes proteins that function in protein aggregation and ROS generation. UCHL1, SYUB (beta-synuclein), SYUA (alpha-synuclein), SEPT5, UBE2K, TPIS, MK01 (mitogen-activated protein kinase 1), GNAO (guanine nucleotide-binding protein G(o) subunit alpha), 1433T (14-3-3 protein theta), 1433E (14-3-3 protein epsilon) and KCRB are associated with the onset of neurodegenerative diseases such as AD and PD (51, 57–63). PP2BA is involved in the formation of Aβ (64). PRDX2, DLDH, CRYM (ketimine reductase mu-crystallin), ALBU (serum albumin), PGAM1, and NDUS3 are related to ROS levels (65, 66). The results demonstrate that when oxidative stress is initiated by chronic restraint stress, ROS levels and proteins involved in protein aggregation increase, and that ROS levels and protein aggregation influence the pathological changes in MDD.

This protein profile study demonstrates that CRS induces many proteome-level changes in proteins functioning in synaptic plasticity, metabolic processes and ROS generation which coordinate to influence the physiological conditions of stressed mice. PTM analysis reveals that Cys90 and Cys152 of UCH-L1 in the hippocampus are oxidized with different sensitivities. Cell survival studies showed that overexpression of UCH-L1 WT or Cys to Asp mutants (C90D, C152D and C90/152D) protects cells from oxidative stress, whereas the Cys to Ser mutants (C90S, C152S and C90/152S) do not. This was not because of the solubility changes of UCH-L1. These results suggest that oxidative modifications of Cys90 (active site of hydrolase) and 152 residues of UCH-L1 confers a protective function in response to oxidative stress and the effect is independent of the hydrolase activity of UCH-L1. C90/152S and C152S seem to act as dominant negatives because of their deteriorating effect on cell viability in response to oxidative stress, indicating that Cys152 particularly play a key role in neuroprotective effect.

UCH-L1, as a deubiquitinating enzyme, which degrades misfolded proteins and recycles ubiquitin molecules under stress condition appears to play a key role in changes in protein profiles and neuroprotection in MDD. However, previous several studies demonstrate the function of UCH-L1 independently of its hydrolase activity (18, 67, 68), because its isoform UCH-L3 has much higher ubiquitin hydrolase activity than UCH-L1 inside cells (67, 69, 70). Both Cys90 and Cy152 of UCH-L1 are evolutionarily conserved in various species, but Cys152 appears only in UCH-L1, not in UCH-L3 (71), suggesting its specific role for UCH-L1 function. Although how Cys oxidations of UCH-L1 protect cells from oxidative stress needs to be fully elucidated by further studies, the possibility can be suggested based on the other antioxidant protein peroxiredoxins, which exerts chaperone function in addition to peroxidase enzymatic activity, as a high molecular weight complex formed in response to oxidative and heat shock stresses (72, 73). A recent study demonstrated the UCH-L1 as a specific client of the peroxiredoxin-2 chaperone (74), which can be correlated with the findings of this study. The solubility differences between Cys to Asp mutants and Cys to Ser mutants of UCH-L1 may also be attributable to this crosstalk between UCH-L1 and Prx chaperone.

Our finding that UCH-L1 protects neuronal cells from oxidative stress, could possibly be used in the prevention of hippocampal atrophy of MDD patients. A previous study has shown that intraperitoneal injection of UCH-L1 protein fused to the transduction domain of HIV-transactivator protein (TAT) into AD model mice improved their contextual memory (14). Cultured primary neurons from UCH-L1 C152A knock-in mice are resistant to 15dPGJ2-induced neurotoxicity (75). This is because of site-specific modification of 15dPGJ2 at Cys152, thereby distinguishing it from the present study in which both Cys90 and Cys152 are affected by oxidative stress. Our 2D-PAGE and PTM analysis indicated that under chronic stress conditions only a small fraction of UCH-L1 retains unmodified Cys90 and Cys152. This is consistent with the findings of Walters *et al.* (70), which showed that total Ub-AMC hydrolase activity remained unaffected in the hippocampus of UCH-L1 deficient mice. Therefore, direct introduction of UCH-L1 WT or activation of UCH-L1 in the brain could be a possible approach to decrease the damage of chronic stress.

In summary, this study demonstrated that UCH-L1 exerts a neuroprotective effect against oxidative stress, in the hippocampus of animals exposed to chronic stress, and that Cys oxidative modifications of UCH-L1 occur from chronic stress. The study also identified the post-translational molecular changes that occur in the brain of animals exposed to chronic stress. This is the first study that highlighted the role of UCH-L1 in the brain under chronic stress as an MDD model. These results might help to understand biochemical changes in the brain of depression patients and have the potential to lead to new therapeutic approaches.

## DATA AVAILABILITY

The mass spectrometry proteomics data for protein identification (Table I) are uploaded into MS-Viewer (UCSF, http://msviewer.ucsf.edu/prospector/cgi-bin/msform.cgi?form=msviewer) (76) repository with the search key sqp0rufe0d. Data are directly accessible via following URL: http://msviewer.ucsf.edu/prospector/cgi-bin/mssearch.cgi?search_name=msviewer&report_title=MS-Viewer&rows_per_page=100&search_key=sqp0rufe0d&.

Raw data for UCH-L1 PTM analyses (Fig. 5, Table II, Table III and supplemental Table S1) have been deposited to the ProteomeXchange (77) Consortium via the PRIDE (78) partner repository with the data set identifier PXD009556.

## Supplementary Material

supplemental Fig. 4

## Footnotes

^1^ The abbreviations used are:

MDD: major depressive disorder
2D-PAGE: two-dimensional PAGE
UPLC: ultra-performance liquid chromatography
1D: one-dimensional
FBS: fetal bovine serum
Pen Strep: penicillin and streptomycin
BCA: bicinchoninic acid
CRS: chronic restraint stress
WT: wild-type
PTMs: post-translational modifications
HBSS: Hanks' Balanced Salt Solution
ROS: reactive oxygen species
siRNA: small interfering RNA
AD: Alzheimer's disease
PD: Parkinson's disease
Ub-AMC: ubiquitin 7-amido-4-methylcoumarin.
