# Supplementary material for: Proteomic Analysis of Hippocampus in a Mouse Model of Depression Reveals Neuroprotective Function of Ubiquitin C-terminal Hydrolase L1 (UCH-L1) via Stress-induced Cysteine Oxidative Modifications
Source: Mol Cell Proteomics. 2018 Jun 29;17(9):1803–23. doi: 10.1074/mcp.RA118.000835 (PMC6126396; doi:10.1074/mcp.RA118.000835)
Supplement: supplemental Fig. 4 [file RA118.000835_index.html]

Supplement to Proteomic Analysis of Hippocampus in a Mouse Model of Depression Reveals Neuroprotective Function of Ubiquitin C-terminal Hydrolase L1 via Stress-induced Cysteine Oxidative Modifications | Molecular & Cellular Proteomics

## Supplemental Data

- supplementary information - Supplementary Experimental procedure Suppl Figure 1-7 Supple Table 1
