# Supplementary material for: Proteomic Analysis of Hippocampus in a Mouse Model of Depression Reveals Neuroprotective Function of Ubiquitin C-terminal Hydrolase L1 (UCH-L1) via Stress-induced Cysteine Oxidative Modifications
Source: Mol Cell Proteomics. 2018 Jun 29;17(9):1803–23. doi: 10.1074/mcp.RA118.000835 (PMC6126396; doi:10.1074/mcp.RA118.000835)
Supplement: supplemental Fig. 4 [file 137938_1_supp_157109_pln5cz.pdf]

Supporting Information

Proteomic Analysis of Hippocampus in Depression Mouse Model Reveals Neuroprotective Function of Ubiquitin C-terminal Hydrolase L1 via Stress-induced Cysteine Oxidative Modifications

Jung-Eun Choi[1], Jae-Jin Lee[1], Wonmo Kang[1], Hyun Jung Kim[1], Jin-Hwan Cho[1], Pyung-Lim Han[2], Kong-Joo Lee[1*]

[1]College of Pharmacy and Graduate School of Pharmaceutical Sciences, and [2]Department of Brain and Cognitive Sciences, Ewha Womans University, Seoul, Korea 03760

*Correspondence: kjl@ewha.ac.kr

Table of contents

Supplementary Experimental Procedures

Supplementary Figure 1
Supplementary Figure 2
Supplementary Table 1
Supplementary Figure 3
Supplementary Figure 4
Supplementary Figure 5
Supplementary Figure 6
Supplementary Figure 7

Supplementary Experimental Procedures

Western analysis

Protein concentration of hippocampal protein samples from control and stressed mice were determined by BCA assay kit (Pierce, USA) and 3 μg of protein per each lane was separated using SDS-PAGE. Proteins were transferred to hydrophobic PDVF membrane (Millipore, USA) and expression level of TPIS, PGAM1, PDXK and GAPDH as a loading control was examined using each primary antibody. Specific antibodies for TPIS, PGAM1 and PDXK were purchased from Abcam (UK), anti-GAPDH was from AbFrontier (Korea). LAS-3000 imaging system (Fujifilm, Japan) and Multi Gauge V3.0 (Fujifilm, Japan) were used to gain chemiluminescent signals and densitometry image analysis. For other 1D-Western analysis in Supplementary Figures, experiments were performed as described in Experimental Procedures. β-actin was used as loading control for soluble/ insoluble fractionation of UCH-L1 in HT-22 cells. β-actin antibody was from Santa Cruz Biotechnology (USA). All Western analysis were conducted under reducing condition.

Identification of overexpressed UCH-L1 spots in HT-22 cells

HT-22 cells were plated at a density of 1 × 10^6 cells/100 mm plate (n=4) and grown for 24 h. 6 μg of DNA plasmids (control plasmid; pcDNA3.1/myc-His(-) A vector, Myc-UCH-L1; control plasmid carrying wild-type (WT) UCH-L1) per each plate were transfected into cells using Lipofectamine 2000 reagent (Invitrogen, USA). Gel strips for 2D-PAGE were rehydrated for overnight. 24 h after transfection, cells were washed twice with 2 mL Hanks' Balanced Salt Solution (HBSS) to remove serum and treated with 0 or 1 mM of H_2O_2 in HBSS for 1 h (37℃, 5% CO_2). Cells were washed twice with 2 mL cold HBSS to completely remove residual H_2O_2. Cells were harvested by scrapping cells with 500 μL of PBS per plate and the cell suspension was centrifuged at 2,500 × g for 3 min at 4℃. Supernatant was removed and the remaining cell pellets were frozen in a -80℃ deep freezer for a while. Pellets were then solubilized using the same lysis buffer for the hippocampus sample preparation by incubating samples at room temperature for 2 h with vortexing at 30 min intervals. The ratio of lysis buffer volume to cell pellet was 100: 12. Samples were centrifuged at 10,000 × g for 20 min at 4℃ to remove cell debris and the supernatant was used for 2D-PAGE. 2D-

PAGE was performed as described in Experimental Procedures. Overexpressed UCH-L1 spots were found by comparing silver-stained gels, and 2D-western in large-gel scale was also carried out to verify the exact distribution of overexpressed myc-UCH-L1. For transfer of proteins to PDVF membrane and further Western analysis, gels were cut into 8 × 10 cm size by comparing with 1: 1 ratio printed image of silver-stained gels, covering the region containing overexpressed UCH-L1 spots. Five major spots were found and the spots were allowed for PTM analysis as described in Experimental Procedures.

Fractionation of soluble and insoluble UCH-L1 in HT-22 cells

To obtain soluble and insoluble fractions of UCH-L1, we followed the method reported by Kabuta *et al.* (Reference 17) with small changes. In brief, HT-22 cells were plated at the density of 5 × 10^5 cells/60 mm plate, grown for 24 h, transfected with 3 μg of DNA per plate (DNA plasmids used in this experiment are same as the plasmids used in xCELLigence RTCA experiments) using Lipofectamine 2000. Cells treated with 0 or 0.3 mM of H_2O_2 in HBSS for 2 h (37℃, 5% CO_2) as previously described, were lysed by scrapping cells with 1% Triton X-100 lysis buffer (250 μL per plate), passed through blue tips for six times, incubated on ice for 15 min. After centrifugation at 20,000 × g for 10 min at 4℃, supernatants were mixed with the same amount of SDS gel sample buffer, boiled for 5 min at 95℃ and this was the nonionic detergent-soluble fraction. Remaining pellets were washed once with lysis buffer, centrifuged at the same condition, and remaining supernatants are clearly removed. 100 μL of SDS gel sample buffer was directly added to the pellet, boiled for 5 min at 95℃ and this was the nonionic detergent-insoluble fraction. As a result, the insoluble fraction is 5 times more concentrated (5x) than the soluble fraction. Soluble and insoluble fractions of UCH-L1 (wild-type and cysteine mutants) were assessed by Western analysis.

Supplementary Figure Legends

Supplementary Figure 1. Representative 2D-PAGE images of hippocampal proteins of control, CRS-9D and CRS-14D mouse.

Supplementary Figure 2. Changes in expression level of TPIS, PGAM1 and PDXK as shown in Figure 3 were verified by Western analysis. (A) Western analysis of hippocampal protein samples from control and stressed mouse (CRS-9D, CRS-14D) showed expression levels of TPIS, PGAM and PDXK. GAPDH was loading control. (B) Densitometry analysis shows quantification results for the blots. Band intensities of each protein was normalized to the intensity of GAPDH. Data were presented as the means ± S.E. of three independent blots (**P<0.01, ***P<0.001).

Supplementary Figure 3. Protein expression levels of endogenous and overexpressed UCH-L1 were detected by Western analysis using anti-UCH-L1 antibody. Samples from Figure 7 were subjected to Western analysis. Tubulin was used as loading control.

Supplementary Figure 4. Identification of overexpressed UCH-L1 spots in HT-22 cells. HT-22 cells were transfected with pcDNA3.1/myc-His(-) A vector and the same vector carrying human UCH-L1 wild-type clone respectively, treated with 0 or 1 mM H_2O_2 and separated by 2D-PAGE. (A) Partial 2D gel images showing UCH-L1 spots overexpressed in HT-22 cells. Five UCH-L1 spots were designated with arrows. (B) Verification of overexpressed UCH-L1 spots using 2D-western analysis. Experiment was performed in large-gel scale.

Supplementary Figure 5. Full 2D-gel images of HT-22 cells overexpressing control myc vector or myc-UCH-L1. Full images of 2D-gels presented in Supplementary Figure 4A shows protein expression profiles of HT-22 cells treated with 0 or 1 mM of H_2O_2. Box indicates the region cropped for Supplementary Figure 4A.

Supplementary Figure 6. Soluble and insoluble fraction of wild-type and Cys mutants of UCH-L1 in response to oxidative stress. HT-22 cells overexpressing WT and Cys to Ser mutants (A) or WT and Cys to Asp mutants (B) of UCH-L1 were exposed to 0 or 0.3 mM H_2O_2 and then soluble and insoluble fractions were separated by cellular fractionation. Control (designated as Cont) means cells transfected with empty pcDNA3.1/myc-His(-) A vector. Proteins from each fraction were analyzed by Western blotting and band intensities of each blot were quantified by densitometry analysis. β-actin was used as loading control. Signal intensity of UCH-L1 was normalized to that of β-

actin. Bar graphs show relative signal intensities of various UCH-L1 constructs compared to the signal intensity of wild-type UCH-L1 treated with 0 mM H_2O_2. Data were shown as the means ± S.E. of triplicates (ns: not significant, *P<0.05, **P<0.01, ***P<0.001).

Supplementary Figure 7. Representative MS/MS spectra of post-translationally modified peptides of UCH-L1 listed in Table 2 and 3 (in the order of appearance in the table).

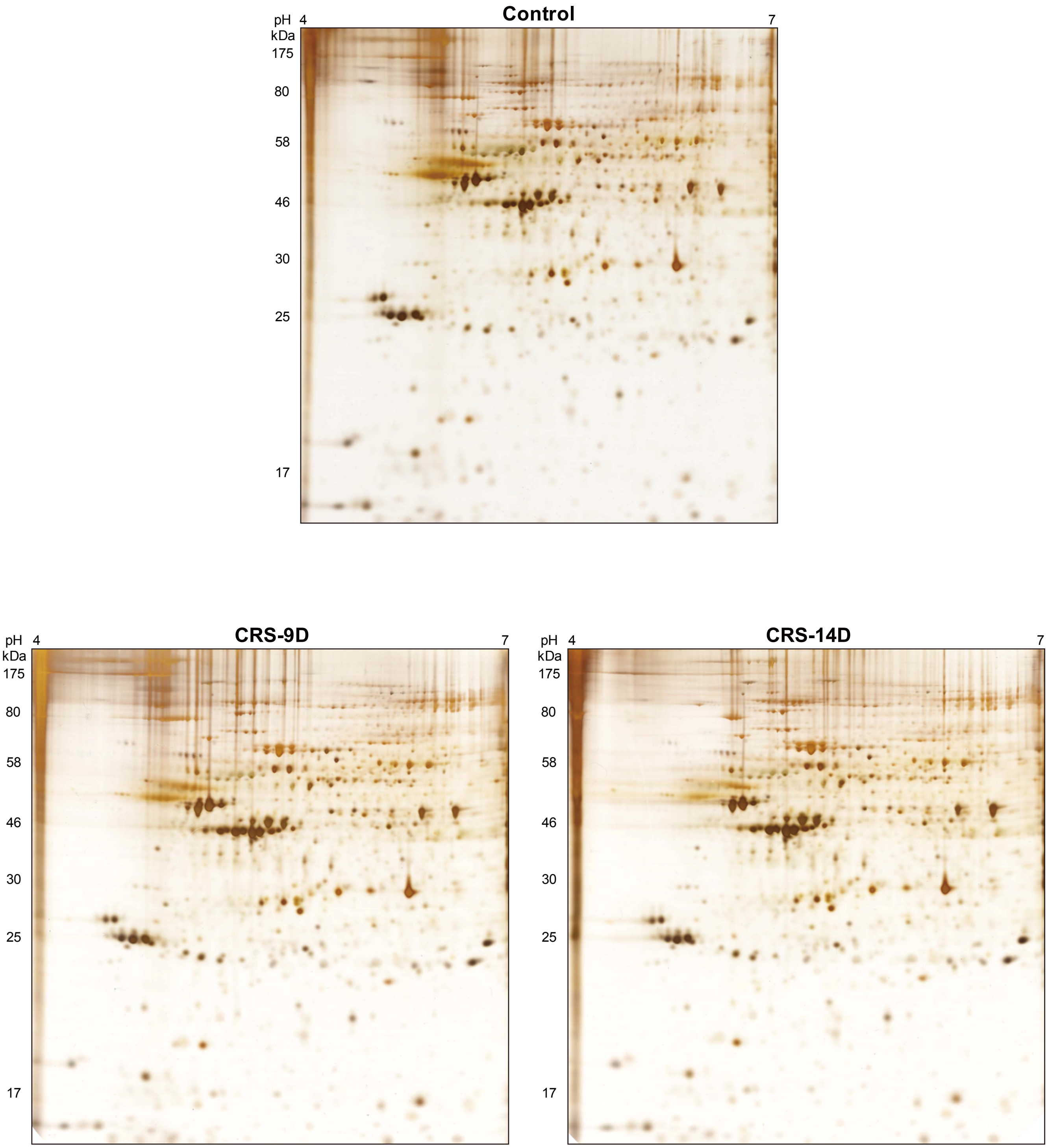

Supplementary Figure 1

Representative 2D-PAGE images of hippocampal proteins of control, CRS-9D and CRS-14D mouse.

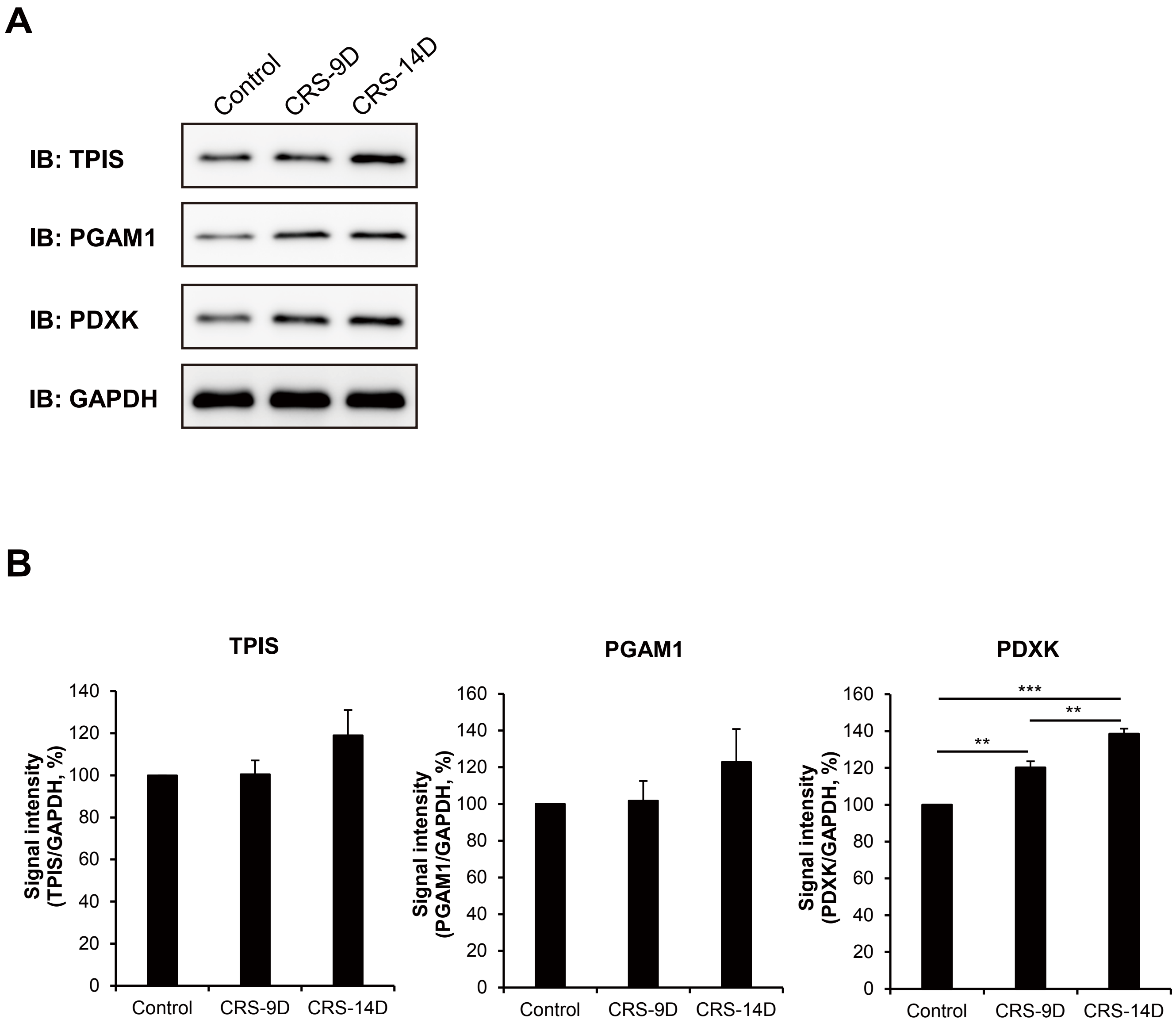

Supplementary Figure 2

Changes in expression level of TPIS, PGAM1 and PDXK as shown in Figure 3 were verified by Western analysis. (A) Western analysis of hippocampal protein samples from control and stressed mouse (CRS-9D, CRS-14D) showed expression levels of TPIS, PGAM and PDXK. GAPDH was loading control. (B) Densitometry analysis shows quantification results for the blots. Band intensities of each protein was normalized to the intensity of GAPDH. Data were presented as the means ± S.E. of three independent blots (**P<0.01, ***P<0.001).

Supplementary Table 1. Ambiguous modifications found in UCH-L1 spots.

Ambiguous post-translational modifications of UCH-L1 spots 1 to 4 in figure 5A were shown in table below. Modifications found in acidic spots were colored in red, those found in basic spots were in blue and modification found in all spots was colored in green.

Start - End	Mass (m/z) Experimental	Mass Observed	Mass Theoretical	Delta Mass (Da)	Sequence / Modification	Spots found
1 - 15	952.1866	1902.3586	1901.8824	0.4763	MQLKPMEINPEMLNK / 2 Acetyl (K); 3 Deamidated (NQ)	Control: 3
1 - 15	635.9644	1904.8714	1904.9045	-0.0332	MQLKPMEINPEMLNK / Acetyl (K); 3 Oxidation (M)	Control: 2, 3<br>Stress: 2, 3, 4
1 - 19	768.3912	2302.1518	2302.1622	-0.0104	MQLKPMEINPEMLNKVLAK / Acetyl (K); 2 Deamidated (NQ); 2 Oxidation (M)	Stress: 4
28 - 64	1042.0044	4163.9885	4164.0530	-0.0645	FADVLGLEEETLGSVPSPACALLLLFPLTAQHENFRK / Carbamidomethyl (C); 2 Deamidated (NQ); Phospho (ST)	Control: 3
65 - 71	465.7620	929.5094	929.5069	0.0025	KQIEELK / Acetyl (K); Deamidated (NQ)	Control: 3<br>Stress: 3, 4
66 - 78	782.8781	1563.7416	1563.7545	-0.0129	QIEELKGQEVSPK / Phospho (ST)	Control: 1, 2, 3<br>Stress: 1, 2, 3, 4
66 - 78	804.8133	1607.6120	1607.7331	-0.1210	QIEELKGQEVSPK / Acetyl (K); 2 Deamidated (NQ); Phospho (ST)	Stress: 1
72 - 83	768.3280	1534.6414	1534.6779	-0.0364	GQEVSPKVYFMK / Acetyl (K); Deamidated (NQ); Phospho (ST)	Stress: 1
79 - 105	775.3681	3097.4433	3097.3803	0.0630	VYFMKQTIGNSCGTIGLIHAVANNQDK / Oxidation (M); Phospho (ST); Phospho (Y)	Stress: 4
79 - 105	1089.9045	3266.6917	3266.5644	0.1273	VYFMKQTIGNSCGTIGLIHAVANNQDK / Acetyl (K); 3 Deamidated (NQ); Oxidation (M); Phospho (ST); Farnesyl (C)	Stress: 2
84 - 105	806.7441	2417.2105	2416.9859	0.2246	QTIGNSCGTIGLIHAVANNQDK / 4 Deamidated (NQ); 2 Phospho (ST)	Control: 3<br>Stress: 2
84 - 115	1140.2069	3417.5989	3417.6133	-0.0144	QTIGNSCGTIGLIHAVANNQDKLEFEDGSVLK / Acetyl (K); 5 Deamidated (NQ)	Control: 2
84 - 115	858.1680	3428.6429	3428.6882	-0.0453	QTIGNSCGTIGLIHAVANNQDKLEFEDGSVLK / Acetyl (K); Oxidation (C)	Control: 1
84 - 115	1170.5688	3508.6846	3508.6545	0.0301	QTIGNSCGTIGLIHAVANNQDKLEFEDGSVLK / Acetyl (K); Phospho (ST); Oxidation (C)	Control: 1
130 - 135	412.7447	823.4748	823.3898	0.0850	AKCFEK / Acetyl (K); Carbamidomethyl (C)	Control: 2
132 - 153	864.3805	2590.1197	2590.1176	0.0021	CFEKNEAIQAAHDSVAQEGQCR / Acetyl (K); 2 Carbamidomethyl (C); Deamidated (NQ)	Control: 2
136 - 153	669.6036	2005.7890	2005.8313	-0.0423	NEAIQAAHDSVAQEGQCR / Phospho (ST)	Control: 1, 2, 3<br>Stress: 1, 2, 3
136 - 153	688.6159	2062.8259	2062.8528	-0.0269	NEAIQAAHDSVAQEGQCR / Carbamidomethyl (C); Phospho (ST)	Control: 1, 2, 3<br>Stress: 2, 3
136 - 153	1027.9089	2053.8032	2053.8160	-0.0128	NEAIQAAHDSVAQEGQCR / Phospho (ST); Trioxidation (C)	Control: 3<br>Stress: 3, 4
136 - 153	666.6362	1996.8868	1996.9021	-0.0153	NEAIQAAHDSVAQEGQCR / Carbamidomethyl (C); Methyl (R)	Control: 2<br>Stress: 1
136 - 153	1040.4309	2078.8472	2078.8364	0.0108	NEAIQAAHDSVAQEGQCR / Carbamidomethyl (C); 2 Deamidated (NQ); Phospho (ST); Methyl (R)	Control: 2
136 - 153	652.6321	1954.8745	1954.8551	0.0194	NEAIQAAHDSVAQEGQCR / Nitrosyl (C)	Control: 2
136 - 153	637.6448	1909.9126	1909.8878	0.0248	NEAIQAAHDSVAQEGQCR / -16 (C)	Control: 2<br>Stress: 3, 4
136 - 153	979.9309	1957.8472	1957.8548	-0.0075	NEAIQAAHDSVAQEGQCR / Dioxidation (C)	Control: 3<br>Stress: 3, 4
179 - 199	1147.4873	2292.9600	2293.0086	-0.0485	MPFPVNHGASSEDSLLQDAAK / Phospho (ST)	Stress: 1, 3, 4
208 - 221	536.8807	1607.6203	1607.7977	-0.1775	EQGEVRFSAVALCK / Carbamidomethyl (C); Deamidated (NQ); Methyl (R)	Stress: 1
214 - 221	488.2601	974.5056	974.4296	0.0760	FSAVALCK / Carbamidomethyl (C); Phospho (ST)	Stress: 1
214 - 221	933.9376	932.9303	933.4031	-0.4728	FSAVALCK / Phospho (ST); Oxidation (C)	spot 2 (pooled)
214 - 223	567.7961	1133.5776	1133.4828	0.0949	FSAVALCKAA / Acetyl (K); Phospho (ST); Dioxidation (C)	spot 3 (pooled)

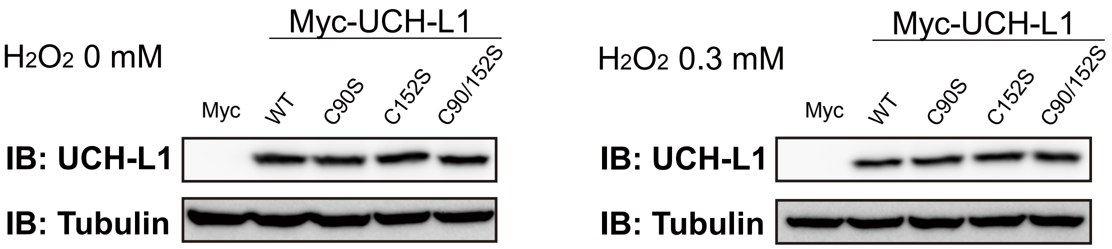

Supplementary Figure 3

Protein expression levels of endogenous and overexpressed UCH-L1 were detected by Western analysis using anti-UCH-L1 antibody. Samples from Figure 7 were subjected to Western analysis. Tubulin was used as loading control.

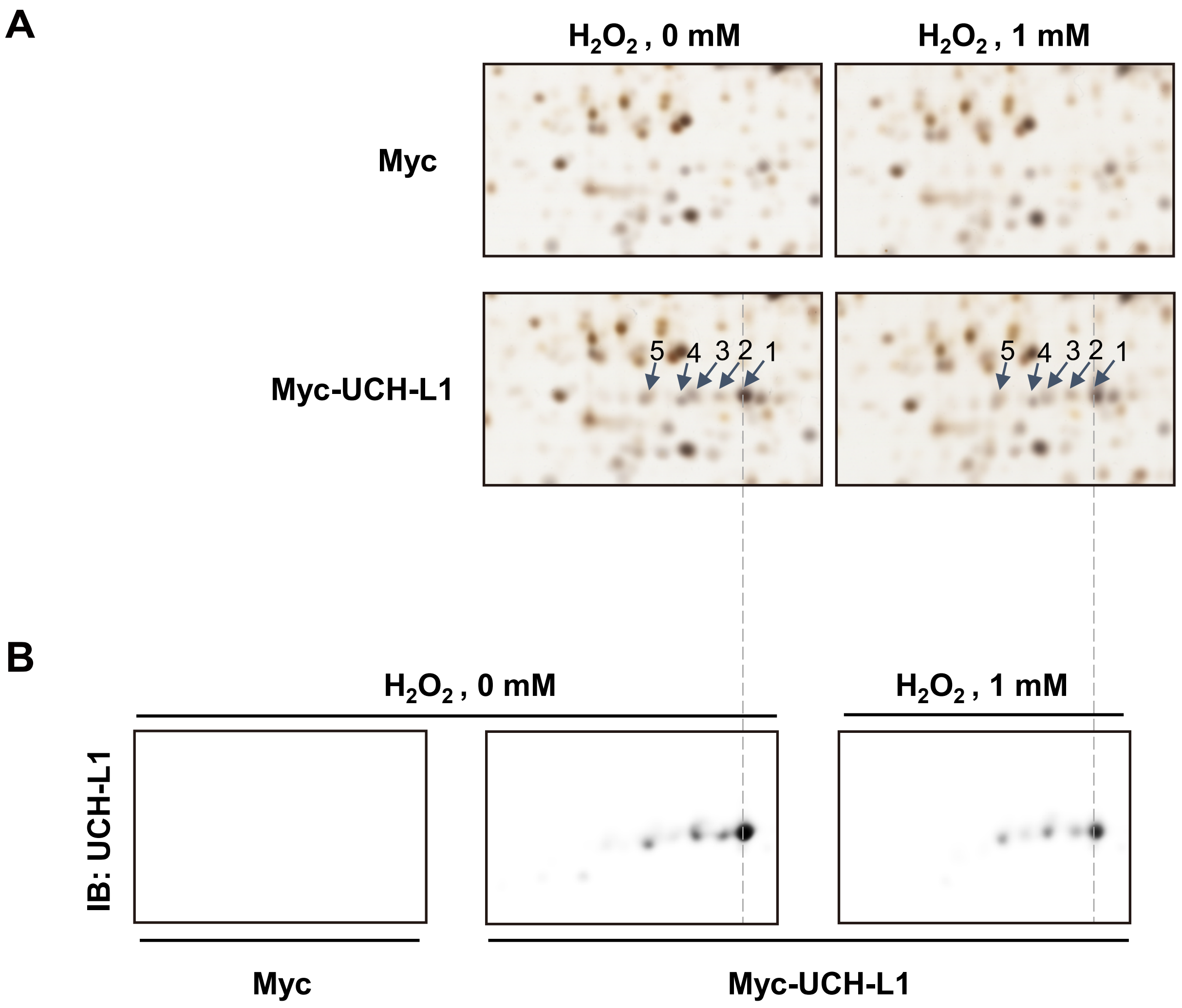

Supplementary Figure 4

Identification of overexpressed UCH-L1 spots in HT-22 cells. HT-22 cells were transfected with pcDNA3.1/myc-His(-) A vector and the same vector carrying human UCH-L1 wild-type clone respectively, treated with 0 or 1 mM H_2O_2 and separated by 2D-PAGE. (A) Partial 2D gel images showing UCH-L1 spots overexpressed in HT-22 cells. Five UCH-L1 spots were designated with arrows. (B) Verification of overexpressed UCH-L1 spots using 2D-western analysis. Experiment was performed in large-gel scale.

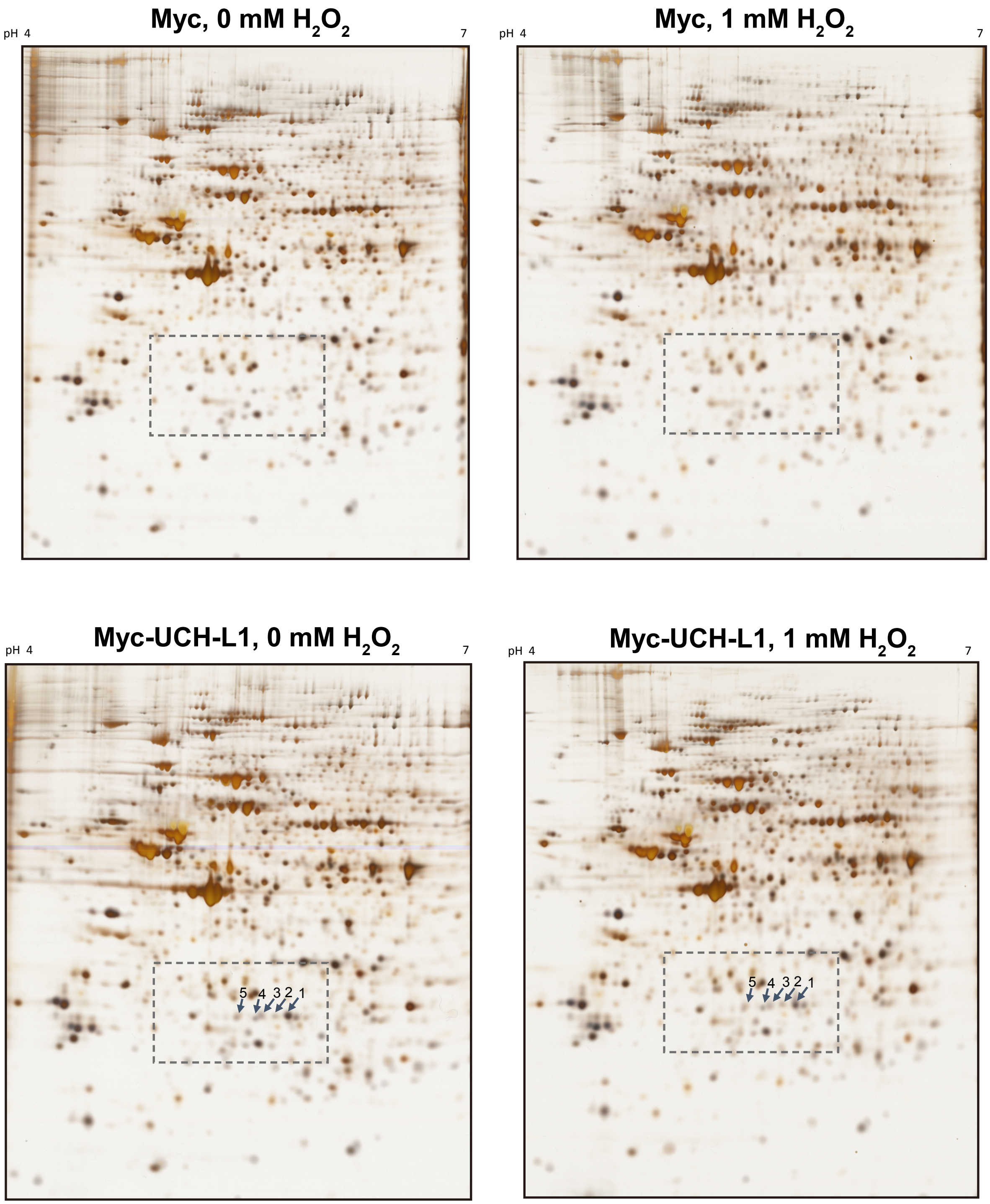

Supplementary Figure 5

Full 2D-gel images of HT-22 cells overexpressing control myc vector or myc-UCH-L1. Full images of 2D-gels presented in Supplementary Figure 4A shows protein expression profiles of HT-22 cells treated with 0 or 1 mM of H_2O_2. Box indicates the region cropped for Supplementary Figure 4A.

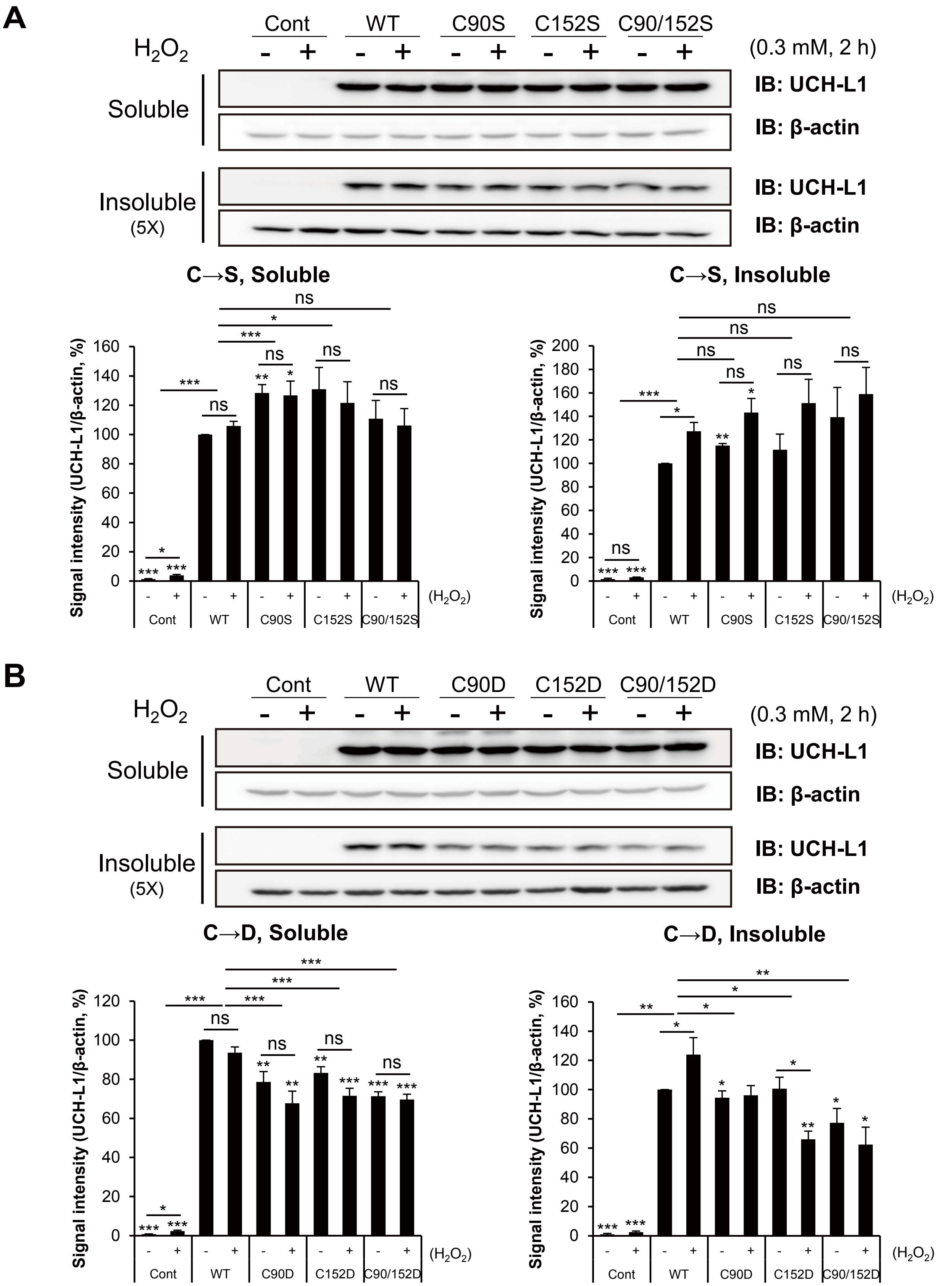

Supplementary Figure 6

Soluble and insoluble fraction of wild-type and Cys mutants of UCH-L1 in response to oxidative stress. HT-22 cells overexpressing WT and Cys to Ser mutants (A) or WT and Cys to Asp mutants (B) of UCH-L1 were exposed to 0 or 0.3 mM H_2O_2 and then soluble and insoluble fractions were separated by cellular fractionation. Control (designated as Cont) means cells transfected with empty pcDNA3.1/myc-His(-) A vector. Proteins from each fraction were analyzed by Western blotting and band intensities of each blot were quantified by densitometry analysis. β-actin was used as loading control. Signal intensity of UCH-L1 was normalized to that of β-actin. Bar graphs show relative signal intensities of various UCH-L1 constructs compared to the signal intensity of wild-type UCH-L1 treated with 0 mM H_2O_2. Data were shown as the means ± S.E. of triplicates (ns: not significant, *P<0.05, **P<0.01, ***P<0.001).

[84]QTIGNSCGTIGLIHAVANNQDKLEFEDGSVLK[115] + Dioxidation (C90)

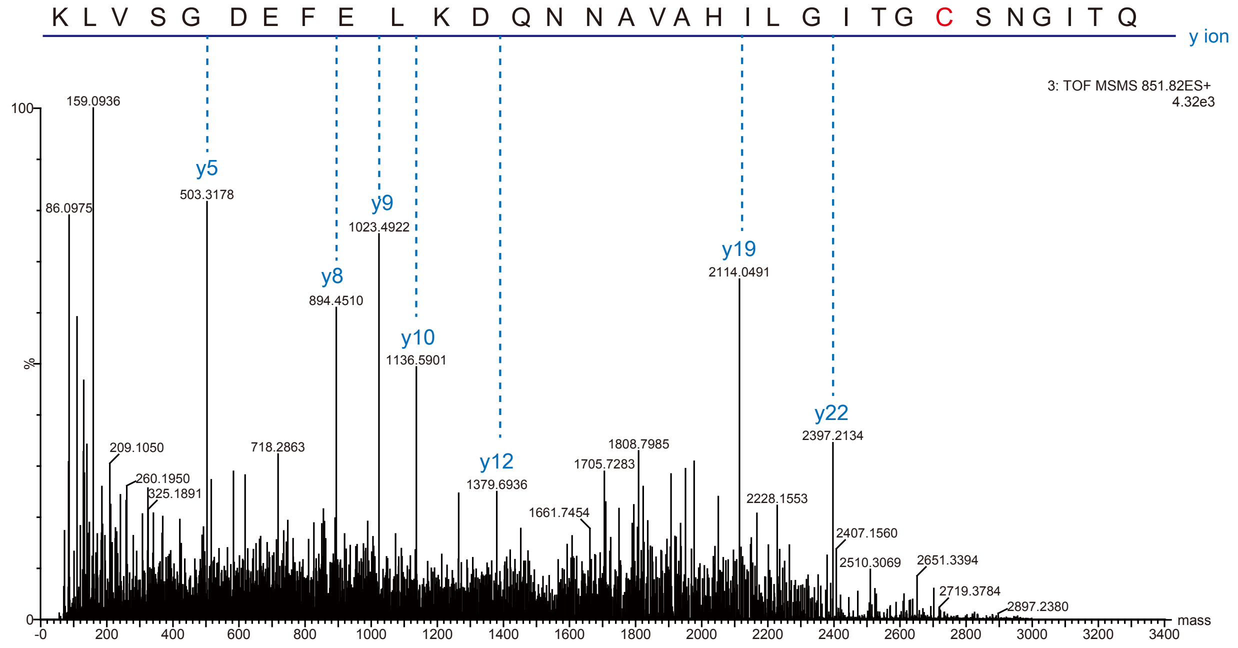

[84]QTIGNSCGTIGLIHAVANNQDKLEFEDGSVLK[115] + Trioxidation (C90)

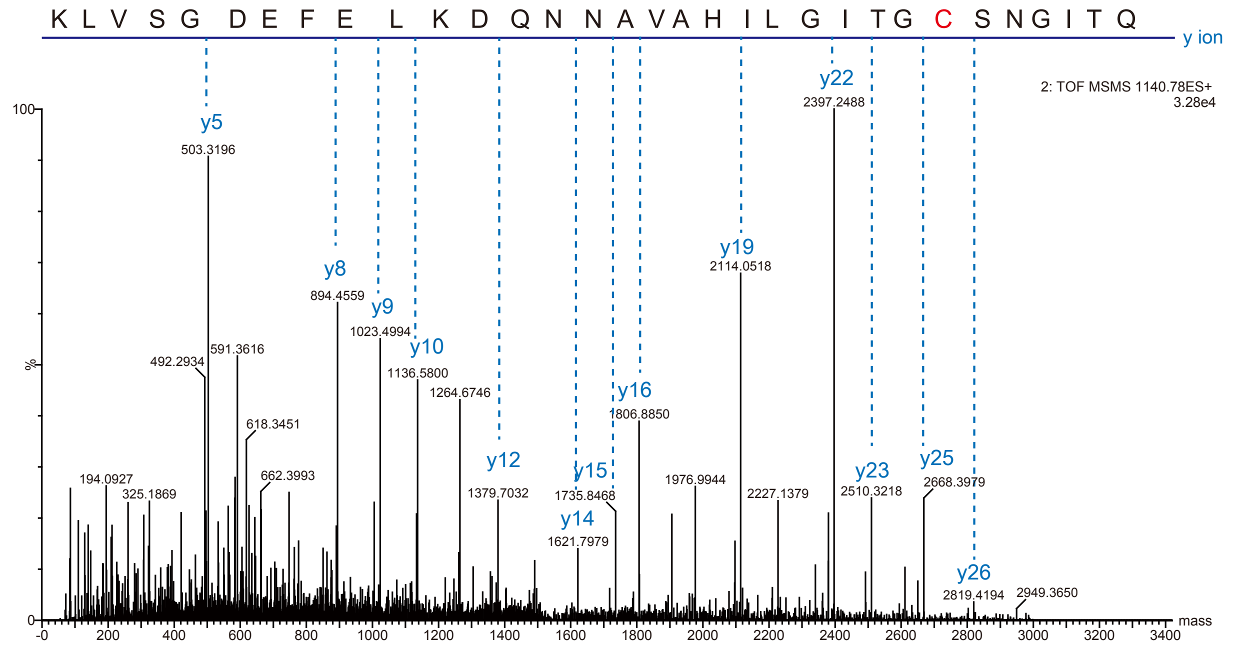

Supplementary Figure 7

Representative MS/MS spectra of post-translationally modified peptides of UCH-L1 listed in Table 2 and 3 (in the order of appearance in the table).

^{84}QTIGNSCGTIGLIHAVANNQDKLEFEDGSVLK^{115} + SO_2-SH (C90)

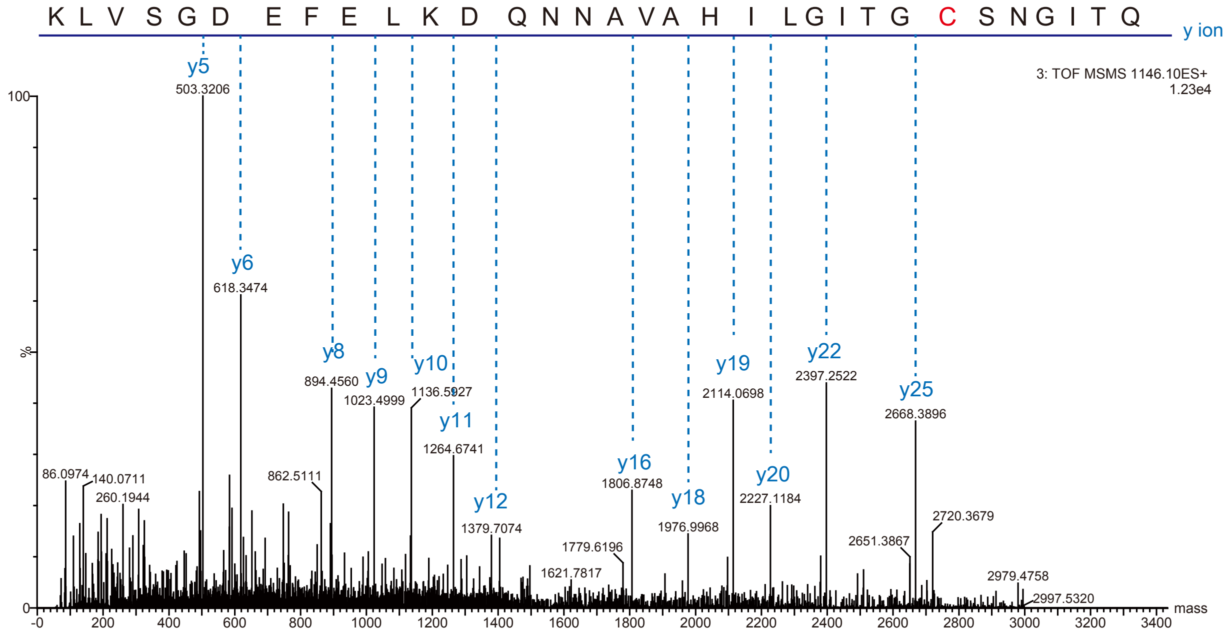

^{136}NEAIQAAHDSVAQEGQCR^{153} + Trioxidation (C152)

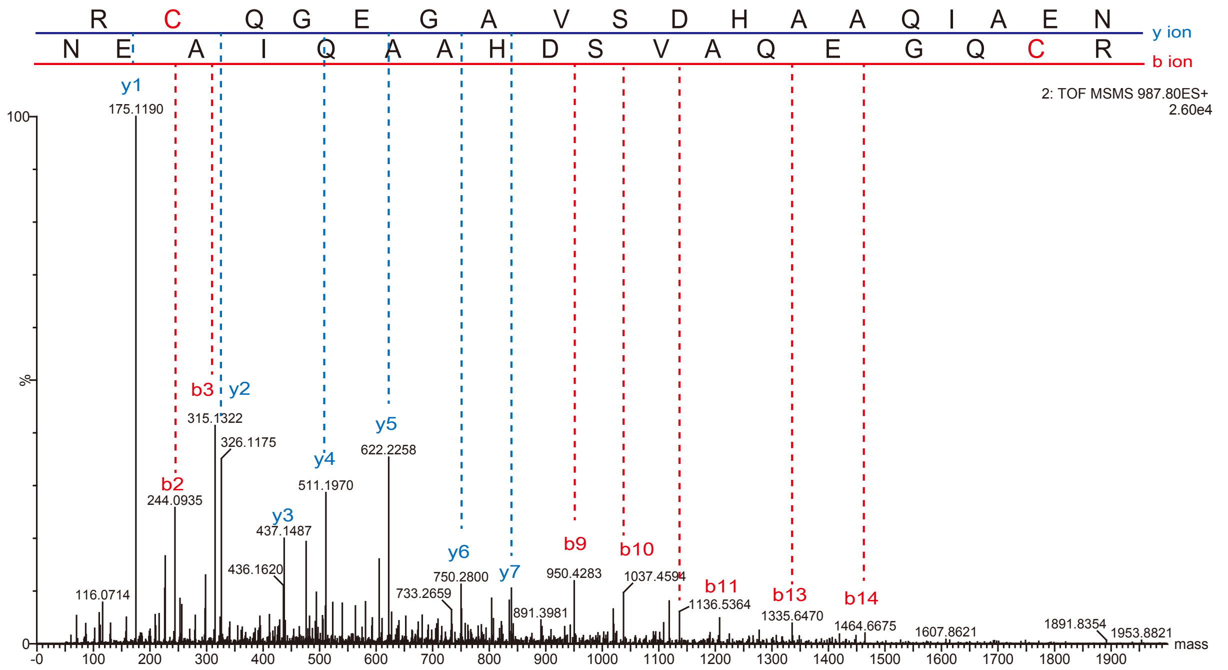

Supplementary Figure 7 (Continued)

^{136}NEAIQAAHDSVAQEGQCR^{153} + SO_2-SH (C152)

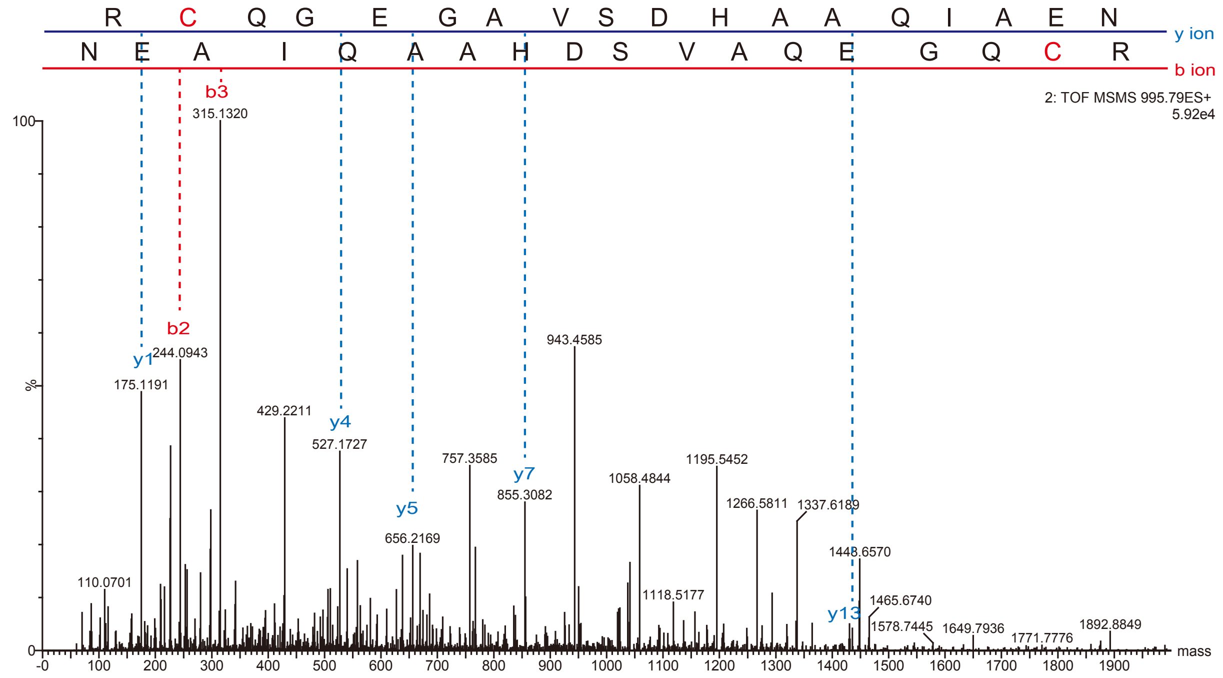

^{84}QTIGNSCGTIGLIHAVANNQDKLEFEDGSVLK^{115} + Phosphorlation (T85), Trioxidation (C90)

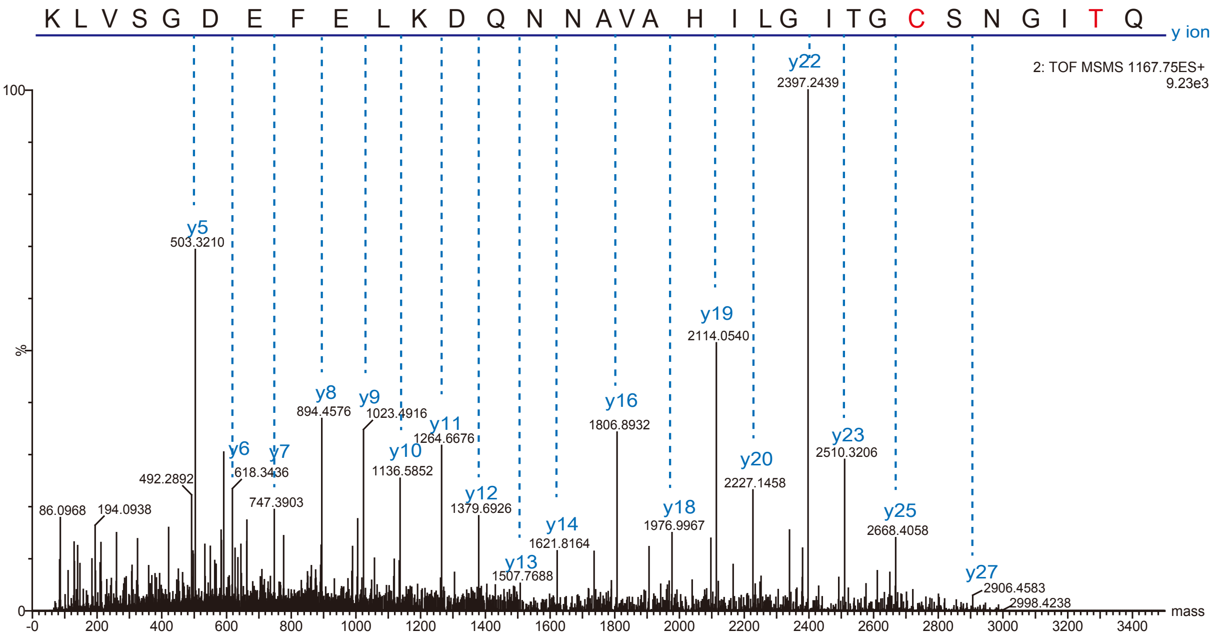

Supplementary Figure 7 (Continued)

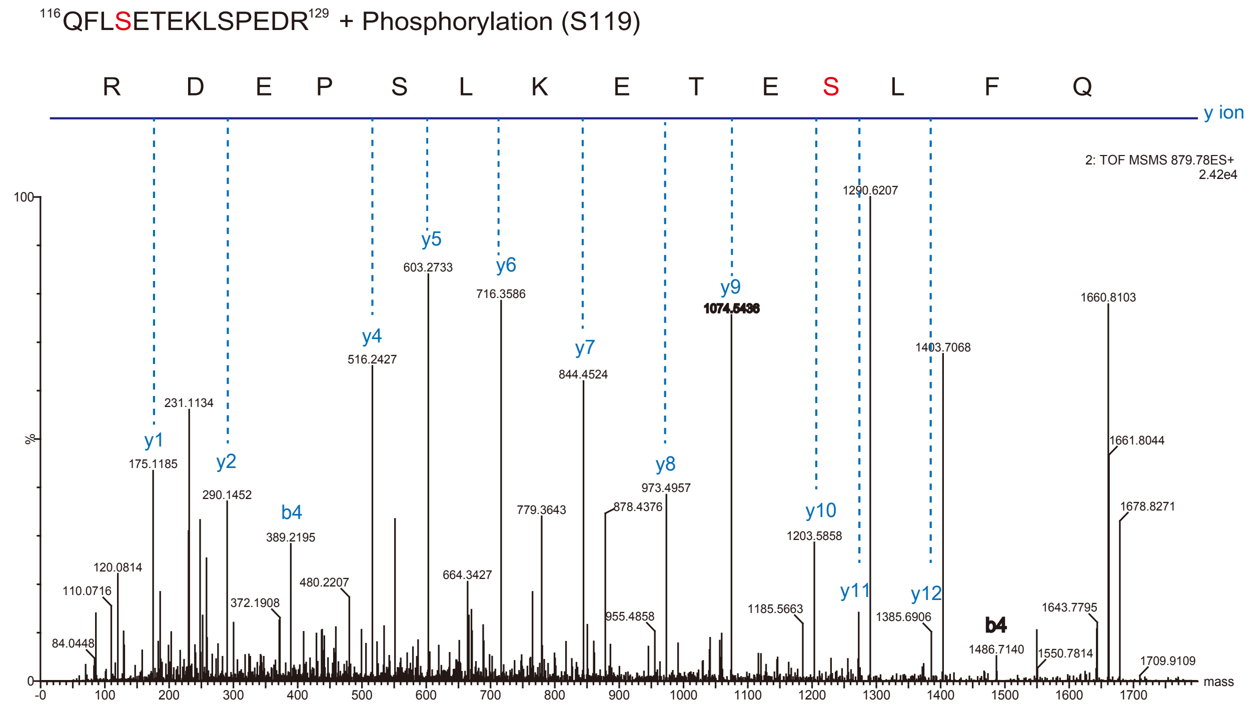

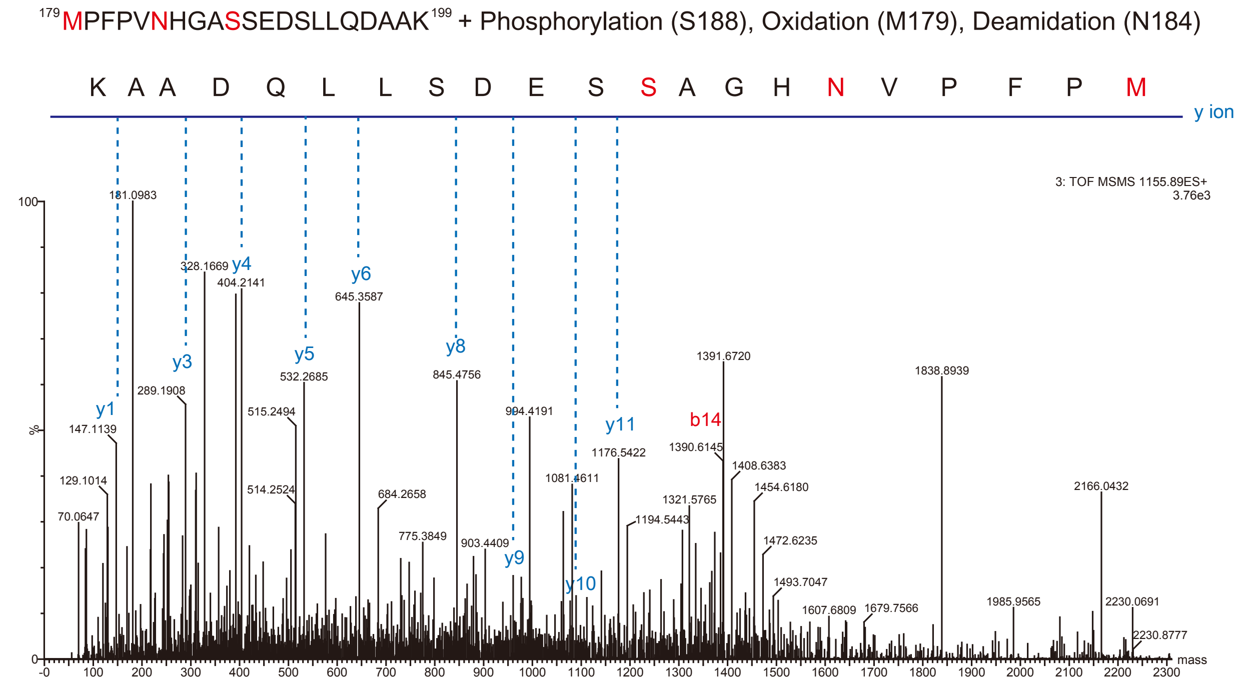

Supplementary Figure 7 (Continued)

66QIEELKGQEVSPK78 + Acetylation (K71), Deamidation (Q66)

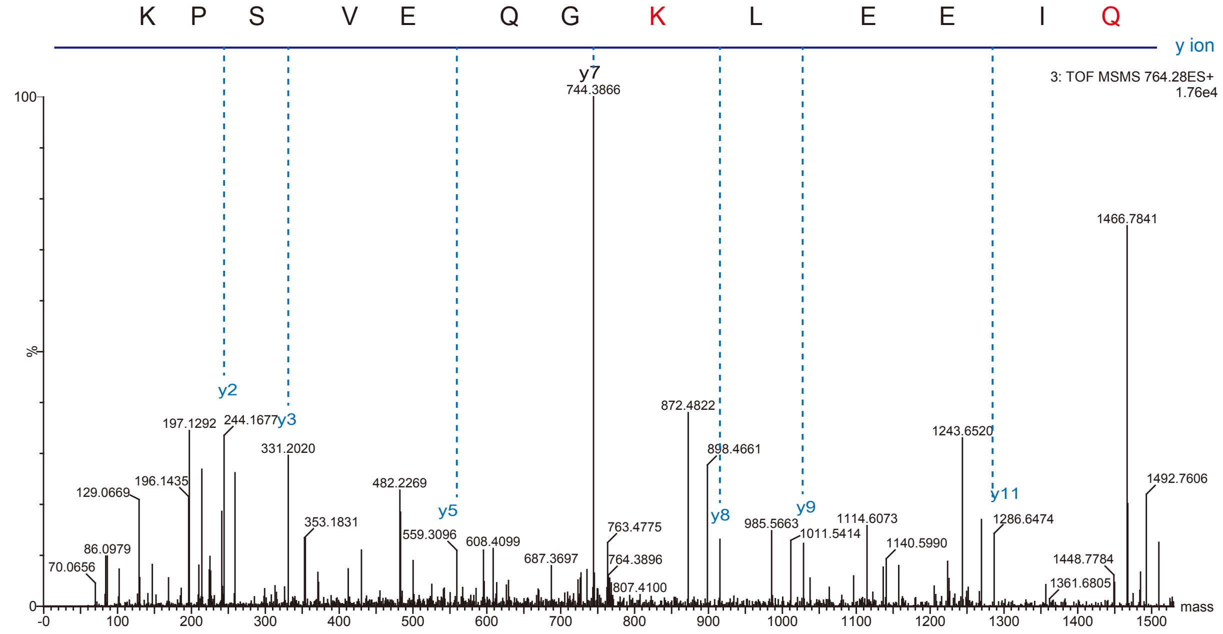

116QFLSETEKLSPEDR129 + Acetylation (K123)

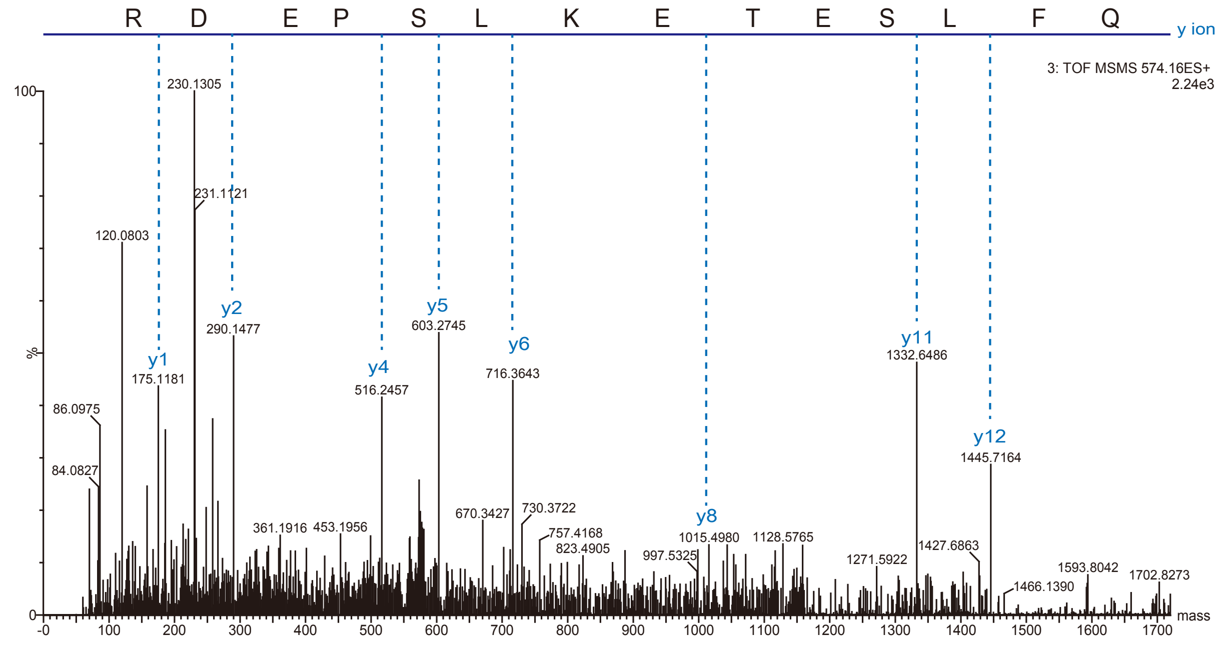

Supplementary Figure 7 (Continued)
